# Synthesis, Structure, and Actual Applications of Double Metal Cyanide Catalysts

**DOI:** 10.3390/ijms251910695

**Published:** 2024-10-04

**Authors:** Ilya E. Nifant’ev, Pavel V. Ivchenko

**Affiliations:** A.V. Topchiev Institute of Petrochemical Synthesis RAS, 29 Leninsky Pr., 119991 Moscow, Russia; phpasha1@yandex.ru

**Keywords:** double metal cyanide complexes, heterogeneous catalysts, Prussian blue analogs, propylene oxide, ring-opening polymerization, carbon dioxide, polyethers, polycarbonates, cyclic carbonates

## Abstract

Double metal cyanide (DMC) complexes represent a unique family of materials with an open framework structure. The main current application of these complexes in chemical industry is their use as catalysts (DMCCs) of the ring-opening polymerization of propylene oxide (PO), yielding branched polyols, highly demanded in production of polyurethanes and surfactants. The actual problem of chemical fixing carbon dioxide from the atmosphere gave new impetus to the development of DMCCs, which turned out to be effective in oxirane/CO_2_ copolymerization. In recent years, new types and formulations of DMCCs were created, so that greater understanding of the reaction mechanisms was achieved and new fields of catalytic applications were found. In the present review, we summarized background and actual information about the synthesis, structure, and mechanisms of the action of DMCCs, as well as their application in the development of new materials and fine chemicals.

## 1. Introduction

Commodity, energy, and environmental concerns drive scientists to develop contemporary approaches to new materials, based on the methods of green chemistry and utilization of wastes and greenhouse gases; improving the efficiency of oil processing is also on the agenda [1,2,3]. Among new-old catalytic systems, widely used earlier for conversion of petroleum-based monomers to in-demand polymer materials and that have received new life in chemical fixation of CO_2_ [4], double metal cyanide (DMC) complexes hold a special place among heterogeneous catalysts [5].

**Scheme 1:**
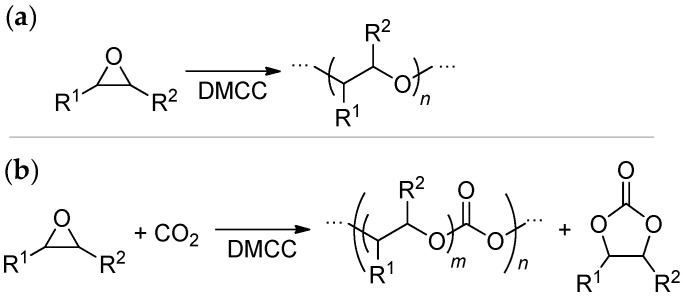
Main catalytic applications of DMCCs: polymerization of oxiranes (**a**) and copolymerization of oxiranes with CO_2_ with a formation of poly(ether-carbonate)s (**b**).

The increased interest in these catalysts arises from ability of DMC complexes to initiate ring-opening polymerization (ROP) of oxiranes (Scheme 1a), mainly propylene oxide (PO), with a formation of polyethers that have multitude of applications ranging from soft components in polyurethane foaming to pharmaceutical formulations [6,7]. The ability of DMC catalysts (DMCCs) to introduce CO_2_ to growing polymer chains with the formation of poly(ether-carbonate)s (PECs) (Scheme 1b) [8,9] maintains interest in these catalytic systems, forcing scientists to continue studies in this field. In recent reviews, the matters of the synthesis, structure, and reaction mechanisms of DMCCs [5,10,11,12,13] and their applications in polymer chemistry [4,5,6,7,8,9,14,15] were discussed. However, the topic of DMC complexes is not exhausted by these aspects, and in the present work we tried to describe and discuss all facets of the preparation, catalytic action, and use of DMCCs, focusing on specific applications, new processes, and materials, based on recent publications in this field.

## 2. Preparation and Structure of DMC Catalysts

DMC catalysts represent heterometallic complexes comprising metal–cyanide and metal–ligand fragments of the formula M_a_[M′(CN)_b_]_c_·xMX_d_·yL·zH_2_O, where M = Zn, Fe(II), Ni(II), etc.; M′ = Co(III), Fe(II), Fe(III), etc.; MX_d_—a salt of metal M, used in the synthesis of DMC complex; and L—organic ligand(s). Stoichiometric factors x, y, and z are determined by the method of preparation as well as type and ratio of the reagents [10,16]. Among DMC complexes, Zn(II)-Co(III) (Zn/Co) systems are the most accessible and widely used. Since 1966, when the use of Zn(II)-Fe(III) (Zn/Fe) and Zn/Fe as a catalysts for polymerization of propylene oxide (PO) was proposed [17], the methods of the preparation of DMCCs were improved, and the chemical nature, composition, and structure of DMC complexes were studied. In scientific periodicals, DMC complexes were first described in 1973 [18], and the first study on optimization of Zn(II)-Fe(III) and Zn(II)-Co(III) catalysts was conducted in the mid-1980s [19]. Comprehensive, reproducible, and reliable results of the studies in the field of DMCCs began to appear in early 2000s, which was due to the progress in physico-chemical methods of the analysis of heterogeneous catalysts and polymers, as well as the development of the theory of catalytic processes with the use of quantum-chemical modeling [20,21,22,23,24,25,26,27,28,29]. In this Section, we present the basic and later results of the studies of preparation and structure of DMC complexes, both widely used Zn/Co systems and prospective bimetallic complexes with different ligands.

### 2.1. Preparation of DMC Complexes

#### 2.1.1. Synthesis of Zn(II)-Co(III) DMCCs

**Scheme 2:**
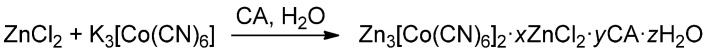
Synthesis of Zn/Co DMC complexes; CA is a complexing agent.

The main method of the preparation of Zn/Co DMC complexes is based on exchange reaction between cyano complexes of Co(III) and Zn(II) salts in the presence of water and (optionally) donor organic ligands (complexing agents, CAs, Scheme 2). Significant excess of Zn(II) salts (usually ZnCl_2_) is critical in the preparation of DMCCs [30], and *^t^*BuOH is widely used as a CA.

A typical synthetic procedure of the preparation of highly active nano-sized Zn/Co DMCCs [20] comprises addition of the aqueous solution of K_3_[Co(CN)_6_] to the aqueous ZnCl_2_ solution with subsequent addition of the solution of *^t^*BuOH and nonionic detergent in water, followed by separation of the reaction product by centrifugation, washing by boiling *^t^*BuOH, and drying. Another order of the mixing of reagent solutions can also be used, e.g., addition of the ZnCl_2_ solution to the solution of *^t^*BuOH and surfactant with subsequent treatment with K_3_[Co(CN)_6_] [23]. An alternative method of the synthesis of Zn/Co DMCCs uses simultaneous addition of ZnCl_2_ and K_3_[Co(CN)_6_] solutions to the solution of CA; this ‘parallel-flow dropping’ method [31] in some cases allows to obtain more efficient catalysts of copolymerization of PO with CO_2_ (see Section 4.2). Active catalysts, prepared using ZnCl_2_ and *^t^*BuOH, have a common formula, Zn_3_[Co(CN)_6_]_2_·x(ZnCl_2_)·y(H_2_O)·z(*^t^*BuOH). These catalysts are raw material intensive; for example, to prepare about 10.0 g of a DMCC, 20.0 g of ZnCl_2_, 8.0 g of K_3_[Co(CN)]_6_, 205 g of water, and 410 g of *^t^*BuOH are needed [32]. To control the reaction, in situ Raman spectroscopy was recently employed. It was shown that at the final stage of the formation of Zn/Co DMCC by the reaction of Zn_3_[Co(CN)_6_]_2_ intermediate with ZnCl_2_ and *^t^*BuOH, the reaction time could be adjusted from hours to minutes through the control of reactant concentrations [33]; however, the further studies on optimization of the Zn/Co DMCC yield are waiting for their researcher. Note that in all early articles [20,21,22,23,24,25,26,27,28], more recent studies [27,34,35], and patents, e.g., [36], the isolated yields of the catalysts were not pointed, making it difficult to compare the efficiency of different methods.

The sequence of mixing of the reagent solutions as well as type and quantity of CA(s) used in preparation of Zn/Co DMCCs directly affect the surface properties of materials. For example, the catalyst prepared by co-precipitation of ZnCl_2_ and K_3_[Co(CN)_6_] solutions in the absence of CAs had a specific surface area (SSA) of 895 m^2^∙g^−1^, pore volume (PV) = 0.592 mL∙g^−1^ and an average pore diameter *d* = 1.3 nm; whereas, depending on the mixing order, reaction temperature, and time, the samples of Zn/Co DMCCs containing *^t^*BuOH and poly(ethylene glycol) (*M*_n_ = 4000, PEG-4000) had SSA = 48.3–486 m^2^∙g^−1^, PV = 0.251–0.518 mL∙g^−1^, and *d* = 2.1–10.3 nm [37].

The use of ZnCl_2_ as a source of Zn^2+^ ions provides higher activity of Zn/Co DMCCs in comparison with the catalysts prepared using other Zn(II) halogenides [27,34,35], Zn(OAc)_2_ [38,39], ZnSO_4_ [38,40,41], or Zn(CF_3_SO_3_)_2_ [40,41]. DMCCs synthesized at higher ZnCl_2_/K_3_[Co(CN)]_6_ ratios demonstrate higher catalytic activity [42]. As was shown by Qi et al. [43], highly active Zn/Co DMCCs can be prepared by the reaction of H_3_[Co(CN)_6_] with Zn(OH)_2_, basic ZnCO_3_, or ZnO in *^t^*BuOH/H_2_O, followed by suspending in aq. HCl and washing by *^t^*BuOH (the yields were not given), but by its nature this catalyst can hardly have been different from ZnCl_2_-based Zn/Co DMCCs. However, simple mixing of H_3_[Co(CN)_6_] and ZnCl_2_ in MeOH resulted in precipitation of [ZnCl]_2_[HCo(CN)_6_]∙MeOH with near quantitative yield [44].

In addition to *^t^*BuOH, different polar organic compounds (e.g., crown ethers [45,46], dicarboxylic acids [47]) and polyethers [20,48,49,50,51,52] were used as co-CAs in Zn/Co DMCC formulations. Organic detergents and dispersants also find use in the preparation of Zn/Co DMCCs; for this purpose, polyacrylamides [53], polyethoxylated sorbitan oleate (Tween^®^ 80) [54], and other reagents [10] were applied.

Besides *^t^*BuOH, many other organic compounds (alcohols, aldehydes, ketones, ethers, esters, carboxylic acids, amides, ureas, nitriles) were tested as CAs [7,10,55]. In an early study [18], the use of Ca_3_[Co(CN)_6_]_2_ was proposed in combination with 1,2-dimethoxyethane; the yield of Zn/Co DMC complex was specified in this work, amounting to 1.05 g of the catalyst per 1 g of the starting Ca_3_[Co(CN)_6_]_2_.

**Scheme 3:**
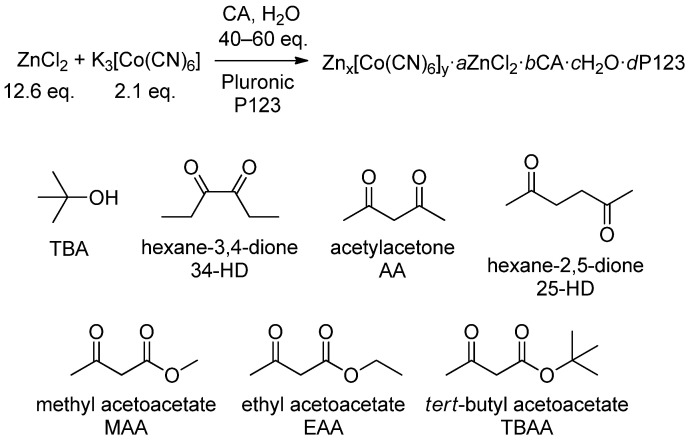
Preparation of Zn/Co DMCCs using different complexing agents [52].

Replacement of *^t^*BuOH by eco-friendly lactate esters in the synthesis of Zn/Co DMCCs was proposed by Kim et al. in 2011 [32] (the yields were not given), the catalysts obtained from HOCHMeCOO^i^Pr and polyethers as CAs were not inferior on productivity to *^t^*BuOH-based DMCCs (see Section 4.1.1). A comparative study of the use of *^t^*BuOH and dicarbonyl compounds of different structure (Scheme 3) in preparation of Zn/Co DMCCs, conducted in 2019 by Kim et al. [52], revealed different types of coordination of CAs (see Section 2.2.1) affecting the activity of the catalysts. The yields of these catalysts were ~1.0 g based on 0.7 g of K_3_[Co(CN)_6_]. The range of dicarbonyl compounds, used as CAs in preparation of Zn/Co DMCCs, was extended in later studies [56,57]; α-, β-, γ-, and δ-dicarbonyl compounds (dicarboxylic acids, dicarboxylic esters, ketoesters and diketones) were investigated, and conclusions about the preference of keto-coordination of CAs for higher catalytic performance in PO polymerization [57] and PO/CO_2_ copolymerization [56] received further experimental proof. Simple and “greener” alternatives to dicarbonyl compounds were cyclic carbonates, proposed by Kim et al. in 2024 to replace *^t^*BuOH in Zn/Co DMCC formulations; the catalysts were prepared in the presence of P-123 as a co-CA, and the yields were not given [58]. In 2024, active Zn/Co DMCCs were also prepared with the use of dialkyl phosphonates, trialkyl phosphites, and trialkyl phosphates as CAs [59]; the yields were not provided.

In 2019, preparation of crystallizable Zn/Co DMCC with the use of Zn(OAc)_2_ instead of ZnCl_2_ was reported [60]. The obtained complex Zn_2_[Co(CN)_6_](OAc)∙4H_2_O comprised positively charged {Zn_2_Co(CN)_6_}^+^ DMC layers linked through OAc groups (XRD data). In some catalytic processes (see Section 4), this complex was not inferior to conventional Zn/Co DMCCs, prepared with the use of *^t^*BuOH.

**Scheme 4:**
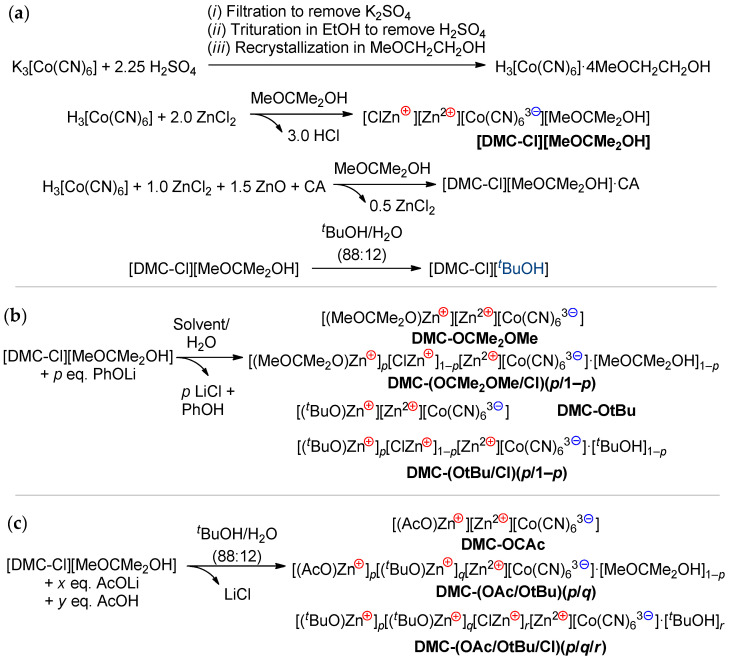
(**a**) Preparation of Zn/Co DMC-Cl catalysts using H_3_[Co(CN)_6_]; (**b**) synthesis of Zn/Co DMCCs containing (RO)Zn^+^ Units and two components of (RO)Zn^+^ and ClZn^+^ units using different complexing agents; (**c**) preparation of Zn/Co DMCCs containing (AcO)Zn^+^ units, two components of (AcO)Zn^+^ and (*^t^*BuO)Zn^+^ units, and three components of (AcO)Zn^+^, (*^t^*BuO)Zn^+^, and ClZn^+^ units [62].

The idea of the use of H_3_[Co(CN)_6_] in the synthesis of Zn/Co DMCCs [43] was painted with new colors when Lee et al. set themselves the task of the preparation of well-defined DMCCs. In 2021, they prepared adducts of H_3_[Co(CN)_6_] with EtOH and MeOH and synthesized a Zn/Co DMC complex by the reaction of H_3_[Co(CN)_6_]∙2.4EtOH (weight form) with Zn(II) 2-ethylhexanoate [61]. In 2024, they synthesized a series of complexes, described by the formula [XZn^+^][Zn^2+^][Co(CN)_6_^3−^] where X = Cl, *^t^*BuO, and/or AcO in different ratios [62]. The methods of preparation of these well-defined Zn/Co DMCCs are presented in Scheme 4.

The use of nitriles [63,64], amides [65], or nitromethane [65] as CAs for Zn/Co DMCCs was studied recently by the scientific group of Kim. The same scientific group also investigated the use of silyl esters as an alternative to *^t^*BuOH [66]. Verma et al. proposed the use of EDTA as a CA (however, complexing properties of this chelating ligand seemed a bit redundant) [67]. The yields of the catalysts were not given in these works.

Low-MW aliphatic amines showed low efficiency as CAs, but higher aliphatic amines demonstrated promising properties when used in preparation of Zn/Co DMCCs [68]. High activity of the catalyst can be attributed to excellent morphology of the catalyst particles, apparently caused by the action of higher amines as surfactants. In [69], cetyltrimethylammonium bromide was used as a CA in the synthesis of Zn/Co DMCC (no yield data) of the selective formation of cyclic carbonates (see Section 4.6).

To reduce solvent and reagent consumption, mechanochemical approaches to Zn/Co DMCCs were examined. In 2011, Zhang et al. proposed a liquid-assisted grinding method for the preparation of Zn/Co DMC complex from ZnCl_2_ and K_3_[Co(CN)_6_] with the use of *^t^*BuOH/decafluoropentane mixture as a grinding liquid (no yield data) [70]. Ball milling was also used for pre-treatment of Zn/Co DMC before its use in preparation of three-component catalyst for copolymerization of PO, CO_2_, and trimellitic anhydride [71] (see Section 4.3).

One of the modern directions of the design of Zn/Co DMCCs is an increase of their surface area via preparation of supported catalysts. In particular, MCM-41-supported catalysts were prepared by simultaneous addition of ZnCl_2_ and K_3_[Co(CN)_6_] solutions to MCM-41 suspensions containing different CAs [72]. Dispersing of Zn/Co DMCC on silica by dry or liquid-assisted grinding was also studied, but a substantial part of the catalyst remained unsupported [73]. More efficient binding of Zn/Co DMCC particles with silica support was achieved when DMC complex was co-precipitated with silica at moderately acidic conditions (sol–gel method) via addition of aq. K_3_[Co(CN)_6_] to an aqueous solution containing ZnCl_2_, Si(OEt)_4_ and HCl, followed by heating until a gel formation, gel separation, drying, and grinding [74]. To increase Lewis acidity and catalytic activity of the DMCCs, a similar method was proposed for the synthesis of Zn/Co DMCCs@TiO_2_ with the use of Ti(OEt)_4_ instead of Si(OEt)_4_ [75]. To increase the catalytic activity of the Zn(OAc)_2_-based layered DMC complex [60], it was supported in situ on Al-Beta-25 zeolite [76].

To sum up, there are two common synthetic approaches to Zn/Co DMCCs. The first approach is based on ion exchange between K_3_[Co(CN)_6_] and ZnCl_2_ in the presence of CA and dispersant. As was shown recently by Chen et al. [33], Zn_3_[Co(CN)_6_]_2_ intermediate is formed rapidly, but further formation of the catalyst takes place over time. Non-stoichiometry of the formation of DMCCs and variability of its composition do not allow a correct comparison of the different methods in terms of the catalyst’s yield. In most cases, the yield of Zn/Co DMCC (in wt%) is roughly equal to the mass of starting K_3_[Co(CN)_6_], but the consumption of ZnCl_2_ (to provide surface saturation of the catalyst by Zn–Cl species) and *^t^*BuOH (to provide its introduction to the catalyst’s framework and elimination of the K^+^ ions) are disproportionately large. The second, more advanced approach to Zn/Co DMCCs is based on the use of K-free cyano complexes of Co [44,62]. This approach provides the formation of Zn/Co DMC complexes with a given composition and high isolated yields, with wide spectrum of catalytic applications. The comparison between the effectiveness of different methods of the preparation of Zn/Co DMCCs is significantly complicated by the absence of the data on the catalyst’s yields in the vast majority of works employing the co-precipitation method.

#### 2.1.2. Synthesis of Zn(II)-Fe(III) DMCCs

Zn/Fe DMC complexes were synthesized and studied in polymerization of PO along with Zn/Co systems back in 1966 [17]. The methods of the preparation of Zn/Fe DMC are similar to those applied in the synthesis of Zn/Co DMCCs, and *^t^*BuOH was widely used as a CA in the preparation of these complexes. In the very recent work of Laguta and van Koningsbruggen [77], the formation of conventional Zn/Fe DMCCs with the use of *^t^*BuOH was studied using the approaches of colloid chemistry. They showed that the catalyst particle at the stage of its formation represents micelle-like species comprising Zn/Fe(CN)_x_ aggregate, decorated by Zn^2+^ ions, and Cl^−^ counterions form the Stern and diffuse layers. Based on the comparison of the Gutmann donor numbers (38 and 33 for *^t^*BuOH and H_2_O, respectively) [77], the role of *^t^*BuOH in the synthesis was to act as a sterically bulky ligand to Zn^2+^, which shields zinc from the complex Fe(CN)_x_ ion.

Pd-doped systems with various amounts of Pluronic P123 co-CA are described in [78]. In the recent study of Kumar et al. [79], Zn/Fe DMCCs with Zn/Fe ratios of 1.49–2.93 were prepared with the use of Na_2_EDTA as a CA, and PEG-200 was added as a co-CA. These catalysts contained minimal amounts of Cl^−^ but retained high catalytic activity in polymerization of oxiranes. As is the case of Zn/Co DMCCs, in most of the publications, the isolated yields of the catalysts were not given.

Relatively recent studies have aimed at finding more material-efficient approaches to these systems. Mechanochemical synthesis of Zn/Fe DMC complexes was performed for the first time by Zhang et al. in 2011 [70], and a simple liquid-assisted grinding method was employed. Solvent-free grinding was used for the preparation of Zn/Fe DMCCs from K_3_[Fe(CN)_6_] and ZnX_2_ (X = Cl, OAc) [39]. Ball milling of the mixtures of K_3_[Fe(CN)_6_], ZnCl_2_, *^t^*BuOH, and different co-CAs was used in preparation of Zn/Fe DMCCs employed in PO/CO_2_ addition and copolymerization [80,81]. The mechanochemical approach to Zn/Fe DMCCs was modified by the use of imidazolium-based ionic liquids, which increased the thermal stability of the catalyst [82]. A highly active catalyst was also obtained by ball milling in the presence of quaternary ammonium salts [83]. The apparent advantages of mechanochemical approaches are high (near quantitative) yields of the catalysts and their known composition. However, when compared with conventional co-precipitation technique, mechanochemical methods provide a lower degree of phase homogeneity.

#### 2.1.3. Synthesis of Other DMC Complexes

Synthetic approaches to other DMC complexes, different from Zn/Co and Zn/Fe(III) systems, are applying similar methods based on ligand exchange. To date, dozens of compounds have been synthesized and studied [84], and the synthesis and structure of similar systems, so-called Prussian blue analogs, have been discussed in recent reviews [11,12,13]. The main field of application of these complexes are the energy storage materials, which is not the subject of our study. In this Section, we will only address some fundamental works in this field and discuss recent publications concerning DMC complexes of interest for the science of catalysis.

A series of Co/M DMCCs were prepared by reacting of aq. CoCl_2_ with K_2_[Ni(CN)_4_], K_4_[Fe(CN)_6_], K_3_[Fe(CN)_6_] or K_3_[Co(CN)_6_] in the presence of *^t^*BuOH and poly(tetramethylene ether) glycol (PTMEG) as the co-CA [85]. Porous DMC complexes Ni_3_[Co(CN)_6_]_2_, Co_3_[Co(CN)_6_]_2_, Fe_3_[Co(CN)_6_]_2_, Ni_3_[Fe(CN)_6_]_2_, Co_3_[Fe(CN)_6_]_2_, and Fe_4_[Fe(CN)_6_]_2_ were prepared by standard co-precipitation method using metal chlorides, hexacyanometallates of Co(III) or Fe (III), and *^t^*BuOH [86]. Hexacyanoferrate(II) derivatives of Zn, Fe(III), Co(II), and Ni(II) were similarly prepared [87]. Co-precipitation of the solutions of K_3_[Co(CN)_6_] and Cu(NO_3_)_2_ was used in the synthesis of Cu/Co DMC complex of the formula Cu_3_[Co(CN)_6_]_2_∙11H_2_O [88].

The synthesis of Zn/Ni DMCCs was conducted by the addition of aq. KCN to the solution of NiCl_2_, followed by treatment with aq. Zn(OAc)_2_ [89]. In [90], ZnCl_2_ was used as a source of Zn for the preparation of this type catalyst. Co/Ni DMCCS were prepared by co-precipitation from K_2_[Ni(CN)_4_] and CoCl_2_ in the presence of *^t^*BuOH and poly(THF) [91].

The complex Co(H_2_O)_2_[Pd(CN)_4_]·4H_2_O, with a clearly defined structure, was obtained by layering a solution of CoCl_2_ in EtOH onto an aqueous solution of K_2_[Pd(CN_4_)], and other Co[M(CN)_4_] complexes (M = Ni, Pt) were synthesized through combination of Co(II) salts with K_2_[M(CN)_4_]; in contrast with Zn/Co DMCCs, preparation of these DMC complexes required complete elimination of Cl^−^, which was a poison for active catalyst [92]. Mechanochemical methods were used in the synthesis of Zn/Cr DMC complexes (starting from K_3_[Cr(CN)_6_], ZnCl_2_ and *^t^*BuOH in 1:10:0.1 molar ratio) [93].

### 2.2. Structure of DMC Complexes

The general structure of DMC complexes includes two metal centers coordinated at C and N atoms of CN^−^ ligands [7,52,94]. Detailed analysis of the structure of DMC complexes is complicated by their low solubility and by strong dependence of their composition and morphology on the method of synthesis. Non-stoichiometric character of active DMCCs [38] adds an additional level of complexity to the determination of the structure of these catalysts and makes it hard to understand the mechanism of the action of DMCCs.

In most works mentioned previously and devoted to the synthesis and use of DMC complexes, their structure and composition is determined by a standard set of methods, including:

- Elemental analysis to determine metal and Cl content;
- Scanning electron microscopy (SEM), including the use of energy-dispersive X-ray spectroscopy (EDX) to determine the size and morphology of the catalyst particles;
- X-ray diffraction (XRD) methods to determine the type of the crystal lattice and degree of crystallinity;
- FT-IR spectroscopy, which gives information about the chemical bonding (M–C≡N^…^M’ fragments, CAs);
- Differential scanning calorimetry and thermogravimetry analysis (DCS/TGA), which reveals the quantity of CAs and the strength of their binding;
- New spectral methods such as extended X-ray fine structure/X-ray near edge structure (EXAFS/XANES) determination that allow determination of the ligand environment of the metal atoms;
- X-ray photoelectron spectroscopy (XPS), for the same purpose.

Also, as was mentioned above, at the stage of DMCC formation, Raman spectroscopy [33] and dynamic light scattering (DLS) [77] are useful and informative.

#### 2.2.1. Structure of Zn/Co DMC Complexes

**Figure 1:**
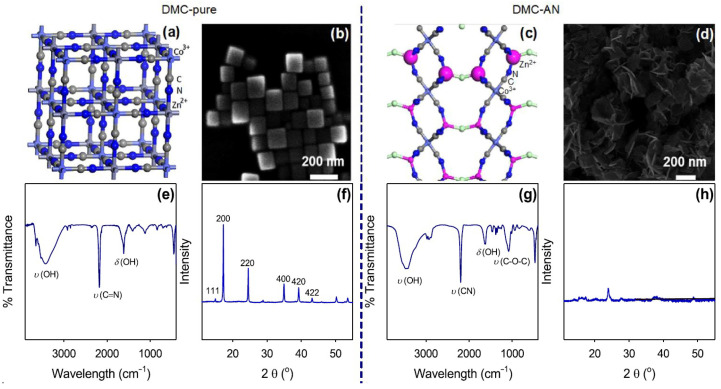
Characterization and comparison of “pure” Zn/Co DMC and Zn/Co DMC-MeCN: (**a**,**c**) DFT-optimized DMC structure. (**b**,**d**) SEM images. (**e**,**g**) FT-IR spectra. (**f**,**h**) XRD patterns. Reprinted with permission from [64]. Copyright (2023) Elsevier B. V., Amsterdam, The Netherlands.

Well-defined structures of crystalline compounds Zn_3_[Co(CN)_6_]_2_ and Zn_3_[Co(CN)_6_]_2_∙12H_2_O [95] have little in common with real structure of Zn/Co DMCCs. However, diversity of non-random vacancy arrangements, found in DMC complexes relatively recently [96], helps to explain high catalytic activity of DMCCs by the ease of mass transfer and defragmentation of catalyst particles observed during polymerization of oxiranes. There is a view that the structure of Zn/Co DMCCs can be considered as a combination of Zn_3_[Co(CN)_6_]_2_ as the backbone and coordinatively unsaturated Zn^2+^ ions with Cl^−^, OH^−^ and ROH ligands on the surface, but this model is too simplistic [6]. Comparative study of Zn/Co DMCCs, prepared without and with CAs, revealed the presence of cubic crystalline phase in the first case, and formation of monoclinic phase in the latter case, also noting that some samples of the catalyst were amorphous [37]. In a recent study by Zhang et al. [97], the formation of a rhombohedral phase was detected in a Zn/Co DMC complex, prepared using *^t^*BuOH as a CA. The visible difference between a “pure” Zn/Co DMC complex and Zn/Co DMCC, prepared using MeCN as a CA, is presented in Figure 1 [64]. The DFT calculations of the face-centered cubic structure of “pure” Zn/Co DMC were based on a cubic F*m*-3*m* symmetry model (Figure 1a). In contrast, for Zn/Co DMC-MeCN, a monoclinic P11*m* symmetry model was used (Figure 1c). These selections were made based on the SEM images showing the crystallite morphologies and the XRD patterns (Figure 1). The level of theory used in DFT optimization is not provided in [64].

**Figure 2:**
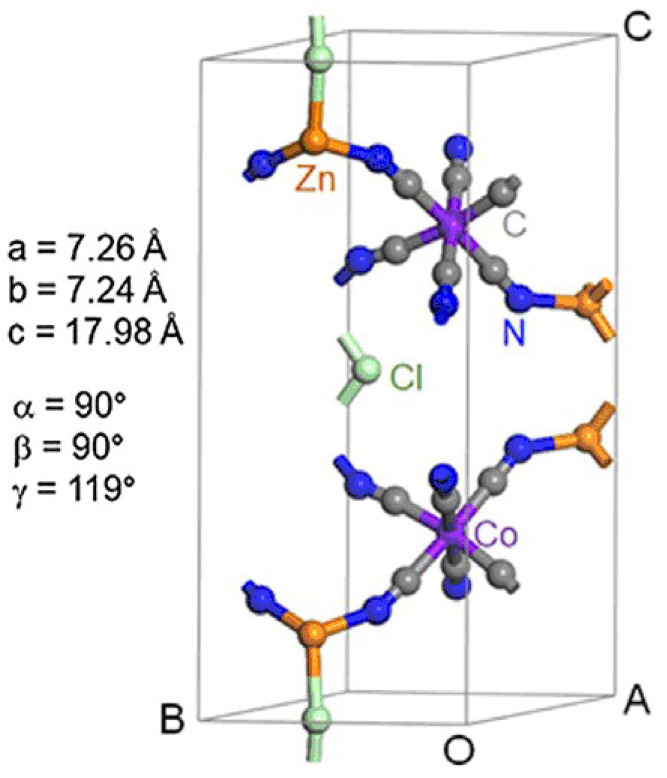
Idealized unit cell of the ZnCl_2_-based Zn/Co DMCC. Reprinted with permission from [52]. Copyright (2019) Elsevier B. V., Amsterdam, The Netherlands.

The idealized structure of Zn/Co DMC complex, containing Cl^−^ ligands (Figure 2), corresponds to the chemical formula Zn_2_[Co(CN)_6_]Cl. In 2007, XRD studies of Zn(II)–Co(III) DMC complexes of different composition showed that pure Zn_3_[Co(CN)_6_]_2_ had a crystallinity of 98.8% with a typical cubic lattice structure, while DMCCs prepared with excess ZnCl_2_ and/or complexing agents had low crystallinity. It is noteworthy that the ZnCl_2_ crystalline phase was not found in DMCCs prepared with an excess of ZnCl_2_ (wide-angle X-ray scattering (WAXD) data), although it seemed that free ZnCl_2_ existed in the catalyst (as evidenced by elemental analysis) [98]. In this work, experimental arguments in favor of Zn_2_[Co(CN)_6_]Cl as a possible base unit of Zn/Co DMCCs were found: the maximum of activity was observed at a 1:1 molar ratio of ZnCl_2_ and Zn_3_[Co(CN)_6_] in DMC complex, which corresponds to Zn_2_[Co(CN)_6_]Cl stoichiometry. Also, it should be noted that when DMC complex Zn_2_[Co(CN)_6_]OH∙4H_2_O was synthesized and studied back in 1987, it had a hexagonal crystal lattice structure [19], but corresponding atomic coordinates, used in further theoretical studies [29,99], were not made public.

**Figure 3:**
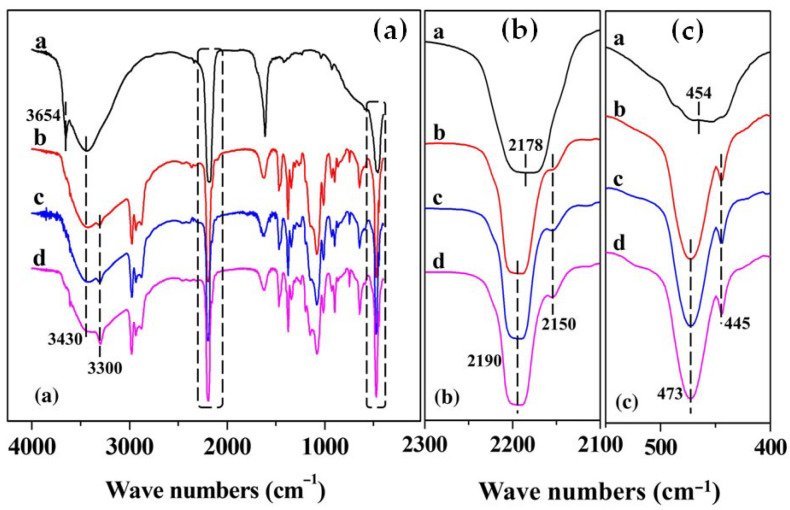
FT-IR spectra of (**a**) Zn–Co DMC catalysts; (**b**) absorption region of –CN; (**c**) absorption region of Co–CN: a—Zn_3_[Co(CN)_6_]_2_⋅nH_2_O, b—DMC-1, c—DMC-2, d—DMC-3. Reprinted with permission from [31]. Copyright (2018) Wiley-VCH Verlag GmbH & Co., Weinheim, Germany.

FT-IR spectral analysis gives some insight into the nature of chemical bonds in Zn/Co DMCCs [31]. The FT-IR spectra exhibit characteristic absorption peaks of –CN, Co–CN and –OH fragments. Stretching vibration of CN^−^ in Zn_3_[Co(CN)_6_]_2_·nH_2_O appeared at 2178 cm^−1^, which is shifted to higher wavenumber in comparison with ν(CN) of K_3_[Co(CN)_6_] (2128 cm^−1^). It proves that CN^−^ acts not only as a σ-donor to Co^3+^ but also as an electron donor in the CN–Zn^2+^ fragment [37]. The shift of ν(Co–CN) band from 416 cm^−1^ for K_3_[Co(CN)_6_] to 454 cm^−1^ for Zn_3_[Co(CN)_6_]_2_·nH_2_O also proves the formation of a Co^3+^–CN–Zn^2+^ structure (Figure 3). In the CN^−^ adsorption region (Figure 3b), additional absorption overlapping peaks (~2190 and 2152 cm^−1^) were detected in the spectra of the Zn/Co DMCCs; these peaks were attributed to amorphous phase and monoclinic structure, respectively. The absorption peaks of Co–CN at 445 and 473 cm^−1^ were assigned to amorphous phase and monoclinic structure, respectively (Figure 3c) [31].

In 2015, a comprehensive study of the structure of Zn/Co DMCCs with the use of EXAFS/XANES determination, powder XRD, EDX, and XPS was conducted by Lawniczak-Jablonska et al. [100]. According to the results of this study, Zn/Co DMCCs, prepared with the use of *^t^*BuOH and excess ZnCl_2_, do not contain ZnCl_2_. EXAFS analysis showed that the coordination around Zn^2+^ was changed from octahedral in reference material Zn_3_[Co(CN)_6_]_2_∙xH_2_O to tetrahedral in catalysts, and Cl atoms were detected near some Zn atoms but no significant amount of O atoms was detected. The Zn atoms inside the clusters had an atomic order similar to anhydrous Zn_3_[Co(CN)_6_]_2_ structure (tetrahedral coordination); the remaining Zn^2+^ ions were defined as bound partially with CN^−^ and with one or two Cl^−^, and located at the surface of the clusters without the formation of ZnCl_2_ phase. The role of CAs is the formation of an amorphous phase, thereby increasing catalytic activity of the complexes [100]. Zn/Co DMCCs, prepared using 1,2-dimethoxyethane (DME), were also studied using EXAFS analysis, and Zn^…^O coordination was not clearly detected [101]. Although EXAFS studies of Zn/Co DMCCs prepared using *^t^*BuOH or DME as CAs have not shown substantial contribution of direct Zn^…^O coordination to the formation of Zn^2+^ ligand environment [102], DSC/TGA have revealed a binding between catalyst matrix and these CAs; it is noteworthy that DMCCs, prepared with the use of *^t^*BuOH, begin to decompose at 120 °C with a formation of isobutylene, which indicates the catalytic character of this process [102,103]. The formation of H_2_O during thermal decomposition of Zn/Co DMCCs may change the nature of the catalyst via the formation of Zn–OH species, this process cannot be ignored in view of the conditions of reactions involving Zn/Co DMCCs. The mechanism of the catalytic action of DMCCs (see Section 3.1) is closely related to the structure of the complexes, and structural experimental data sometimes contradict hypothetical structures used as the basis for quantum-chemical modeling.

**Figure 4:**
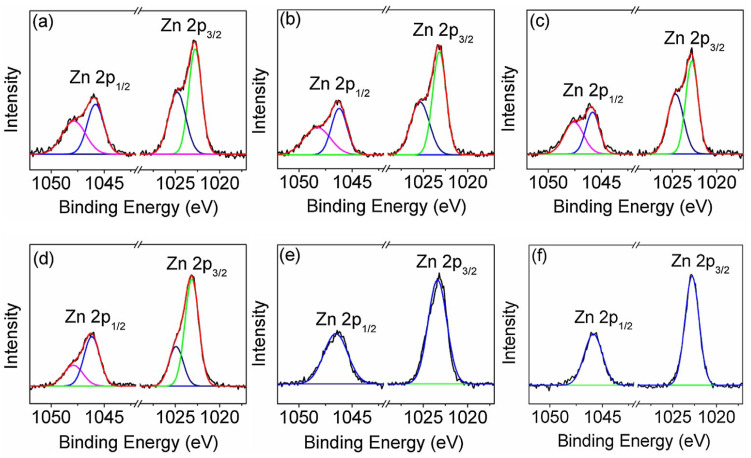
Expanded Zn 2p XPS spectra of various DMC catalysts: (**a**) DMC-AA prepared at 50 °C, (**b**) DMC-MAA prepared at 50 °C, (**c**) DMC-EAA prepared at 90 °C, (**d**) DMC-TBAA prepared at 70 °C, (**e**) DMC-34HD prepared at 50 °C, and (**f**) DMC-25HD prepared at 50 °C. For CA abbreviations, see Scheme 3. Reprinted with permission from [52]. Copyright (2019) Elsevier B. V., Amsterdam, The Netherlands.

Comparative study of Zn/Co DMCCs, prepared using different dicarbonyl compounds as CAs (Scheme 3), revealed different types of coordination of CAs at Zn^2+^ ion [52]. These types of coordination were detected using FT-IR spectroscopy; clear evidence of difference in Zn^2+^ bonding with α-, β-, and γ-diketones and β-ketoesters was visible based on analysis of expanded X-ray photoelectron spectra (XPS) of DMCCs (Figure 4). The Zn 2p_1/2_ and Zn 2p_3/2_ spin-orbital peaks are usually ascribed to two singlets at 1044–1046 eV and 1021–1023 eV. The XPS spectra of Zn 2p in the case of DMC-AA, DMC-MAA, DMC-EAA, and DMC-TBAA (see Scheme 3) exhibited two doublets of Zn 2p_1/2_ and Zn 2p_3/2_. The splitting of spin-orbital peaks resulted from Zn^…^O coordination. The XPS spectra clearly indicated that β-dicarbonyl CAs coordinate to the Zn^2+^ in either a keto or enol fashion. For DMC-34HD and DMC-25HD (see Scheme 3), the XPS spectra of the Zn 2p_1/2_ and Zn 2p_3/2_ spin-orbits were deconvoluted into two singlets (1022.9/1046.1 eV and 1023.4/1046.3 eV, respectively), indicating the presence of the only Zn^…^O coordination mode. These experimental observations were confirmed by the results of the DFT modeling of CA’s coordination on the surface of Zn/Co DMCC (CASTEP module in BIOVIA Materials Studio 5.0 software package, Perdew–Burke–Ernzerhof (PBE) functional).

**Figure 5:**
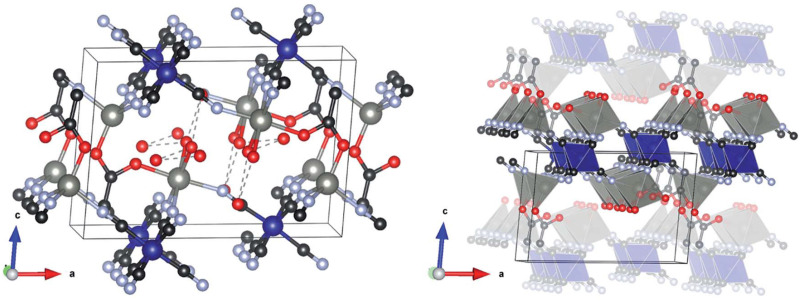
Crystal structure of Zn_2_[Co(CN)_6_](OAc)∙4H_2_O (**left**) and polyhedral view showing the stacking of the layers in Zn_2_[Co(CN)_6_](OAc)∙4H_2_O (**right**). Red, blue, light grey, dark grey, and black spheres represent O, N, C, Zn, and Co atoms, respectively. H atoms were omitted for clarity. Reprinted with permission from [60]. Copyright (2019) Royal Society of Chemistry, London, UK.

Although the structures of the vast majority of Zn/Co DMCCs, both laboratory and industrially implemented, were not clearly confirmed by XRD analysis, XRD studies were successful for some complexes. The structure of the previously mentioned Zn_2_[Co(CN)_6_]OH·4H_2_O [19] served as the basis for subsequent theoretical investigations (see below). The complex Zn_2_[Co(CN)_6_](OAc)·4H_2_O, crystallized in the monoclinic space group *P*2_1_/*m*, had a layered structure (Figure 5); the asymmetric unit consisted of two symmetry independent Zn atoms and one Co atom. Zn1 was tetrahedrally coordinated to three N atoms and one O atom of the OAc^−^. Zn2 had an octahedral coordination consisting of three N atoms, one O atom from an OAc^−^ group, and two water molecules [60].

**Figure 6:**
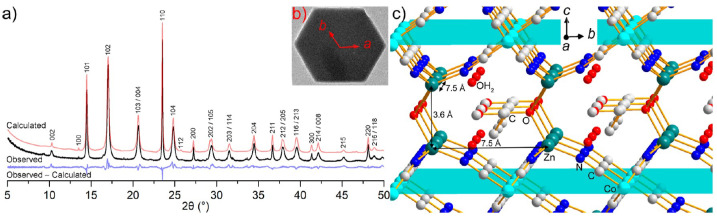
(**a**) Rietveld refinement of synchrotron XRD data, (**b**) TEM image of a hexagon plate, and (**c**) elucidated structure of [(MeOCMe_2_O)Zn^+^][Zn^2+^][(Co(CN)_6_^3−^]. In (**c**), only the MeOCMe_2_O-fragment is depicted among the three disordered positions, with hydrogen atoms omitted for clarity. Reprinted with permission from [62]. Copyright (2024) American Chemical Society, Washington, D.C., USA.

The structure of [(MeOCMe_2_O)Zn^+^][Zn^2+^][Co(CN)_6_^3−^] was also clearly established by XRD methods [62]. The complex crystallized in hexagonal space group *P6*_3_/*mmc*, the overall structure represented a stack of layers, with each layer characterized by the composition of [(MeOCMe_2_O)Zn^+^][Zn^2+^][Co(CN)_6_^3−^] (Figure 6). The interlayer distance was 8.5 Å, thereby creating channels and cavities between layers. These channels and cavities host approximately two H_2_O molecules (1.75H_2_O) as well as a MeOCMe_2_O-fragment per [(MeOCMe_2_O)Zn^+^][Zn^2+^][Co(CN)_6_^3−^] unit.

It should be noted that the Zn_2_[Co(CN)_6_]X structural motif was used and can still be used for mechanistic analysis of chemical behavior of Zn/Co DMCCs in different catalytic processes. The first attempt of density functional theory (DFT) modeling of the active sites in Zn/Co DMCCs (PW91 exchange and correlation functional for periodic calculations, B3LYP/LANL2DZ level of theory for molecular optimizations) was made by Wojdeł et al. in 2007 [29]. The aim of this study was to select an atomic cluster adequately representing the active site of the DMC catalyst. Calculations were made for the cluster model containing six atoms of Co and twelve atoms of Zn; this model was based on unpublished XRD data obtained previously for Zn_2_[Co(CN)_6_]OH·4H_2_O. B3LYP/LANL2DZ level of theory, used in this work, raises certain questions; the values of Bader charges at C and O atoms (1.92–1.93 and −2.45, respectively) are of interest. The next attempt of quantum-chemical modeling of the structure of Zn/Co DMCC took place in 2015: López et al. took the native DMC structure (hexagonal unit cell, in which the octahedral Co unit acts as a spacer for the tetrahedral Zn polygons) to reoptimize the cells using PBE functional [99]. The authors chose to fully replace Cl anions in the lattice by OH, OEt, and O*^t^*Bu ligands, with subsequent evaluation of the sites obtained in catalytic process.

**Figure 7:**
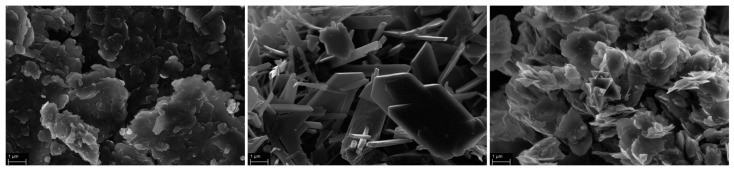
SEM images enlarged by a magnitude of 20,000 of different Zn/Co DMCCs that were gifts from industry. Reprinted with permission from [104]. Copyright (2024) Royal Society of Chemistry, London, UK.

In conclusion, it should be noted that the current industry uses different types of Zn/Co DMCCs, prepared from ZnCl_2_ and K_3_[Co(CN)_6_] in various reaction conditions (solvent, CAs, temperature, concentration (gradients), washing and drying procedures), which typically leads to divergent morphologies and crystallinities (Figure 7) as well as catalytic characteristics [104].

#### 2.2.2. Structure of Other DMC Complexes

**Figure 8:**
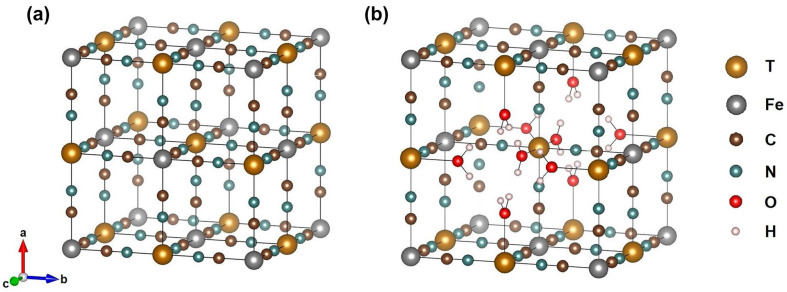
Crystal network of transition metal hexacyanoferrate(II) complexes without [Fe(CN)_6_]^3−^ vacancies (**a**) and with [Fe(CN)_6_]^3−^ vacancies (**b**). Reprinted with permission from [87]. Copyright (2022) Springer Nature, London, UK.

Divalent metal (M) hexacyanometallates M_x_[M’(CN)_6_]_2_) are, by far, the most studied class of cyanometallate compounds. They usually crystallize in an open microporous cubic structure (Figure 8a). When the negative charge of the hexacyanometallate ([M’(CN)_6_]^b−^) block and the positive charge of the divalent transition metal cation M^2+^ differ, hexacyanometallates do not meet the oxidation state sum rule. Instead, some molecular [M(CN)_6_]^b−^ blocks are randomly vacant in the structure (Figure 8b), providing formation of open metal sites with active M^2+^ centers. Comparative study of several Prussian blue analogs M_3_[M’(CN)_6_]_2_ (M = Fe, Co, Ni, Mn, Cu; M’ = Fe, Co) revealed proximity in structure and the presence of small and large pores [105].

The powder XRD pattern reported by Zhang et al. [39] of a Zn/Fe DMC obtained by ball milling of K_3_[Fe(CN)_6_] and Zn(OAc)_2_ was qualitatively similar to the diffraction pattern of Zn_2_[Co(CN)_6_](OAc) [60], and the layered structure can be proposed for this type of Zn/Fe DMC catalyst. The layered structure of the complexes M[Ni(CN)_4_] (M = Ni, Co, Fe, Mn), providing their high catalytic activity, was revealed recently by Penche et al. [106].

## 3. Mechanistic Insights of the Action of DMC Catalysts

### 3.1. DMC Catalysts in Polymerization of Oxiranes

**Scheme 5:**
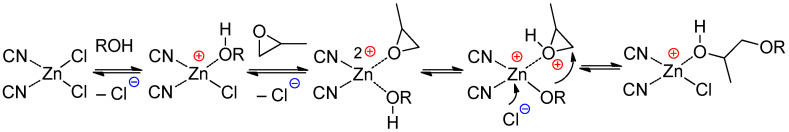
Coordinative cationic polymerization mechanism for DMC-catalyzed ROP of PO (initiation stage) proposed in early works [38,98].

As was showed experimentally in the earlier works [38,98], Zn_3_[Co(CN)_6_]_2_ has low activity, as well as DMC complexes prepared without excess of ZnCl_2_ even in the presence of CAs, whereas excess of ZnCl_2_ endows DMCCs with high activity even in the absence of CAs. The mechanism of initiation of PO polymerization, proposed in [38,98], is presented in Scheme 5. In [107], a very similar mechanism with a replacement of both Cl^−^ ligands by ROH was presented; this mechanism was mentioned in review of Herzberger et al. [7], despite the low probability of double substitution of Cl^−^ ligands by ROH (quite apart from the fact that the presence of ZnCl_2_ fragment in DMCC is doubtful, see Figure 1 and Figure 2).

**Figure 9:**
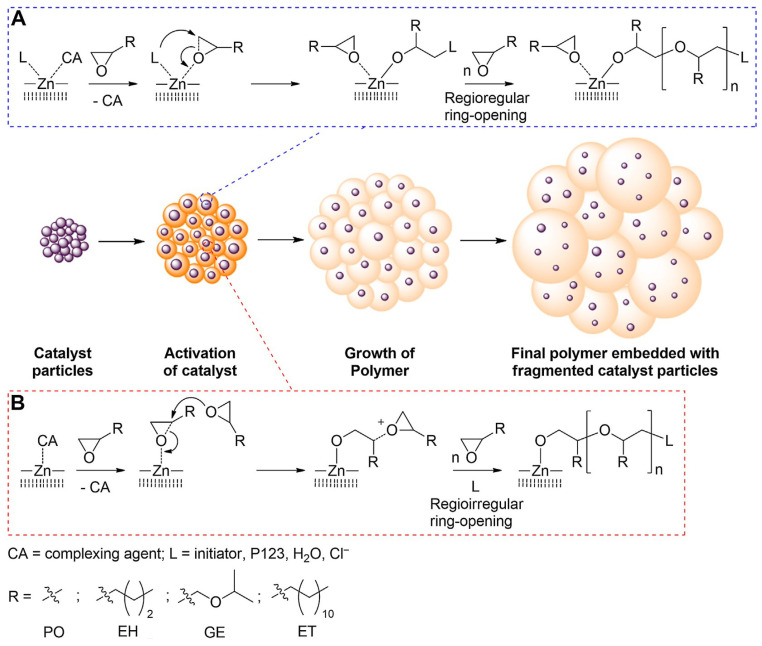
Proposed mechanistic pathways of DMC-catalyzed polymerization of oxiranes. The initiation proceeds via both coordinative route at the catalyst surface (**A**) and activated-chain end route at the interstitial site (**B**), resulting in fragmentation of the catalyst particles. Reprinted with permission from [52]. Copyright (2019) Elsevier B. V., Amsterdam, The Netherlands.

In comparative experimental and theoretical studies of Zn/Co DMCCs, prepared with the use of CAs of different nature (*^t^*BuOH and dicarbonyl compounds) [52], the mechanism of polymerization of oxiranes was studied in more detail. The authors took into account the results of the study of Prussian blue analogs [105] that revealed two types of micropores located within the DMC framework: small pores formed by the defect-free unit cells and larger pores formed by the one-third random vacancies, as required by electrical neutrality. Bearing in mind estimation of the pore size in Zn-Fe/Co DMC complexes (~6.1 Å) [108], they proposed that PO monomers are too small to pass through the pores of various sizes (apparently, due to amorphous nature and the presence of small molecules eliminated during activation, real DMCCs contain pores of larger diameter). Once the monomers adsorbed in the interstitial sites are activated, the growing polymer chains induce stress to the catalyst matrix, followed by fragmentation of the catalyst particles, thereby exposing active interstitial sites. The phase changes observed during polymerization included fine dispersion of the catalyst particles at early stage, and this process was accompanied by the loss of Zn^…^CA coordination. Defragmentation the catalyst particles was confirmed experimentally by dynamic light scattering (DLS) measurements [52]. The polymerization mechanism proposed in [52] is presented in Figure 9. At the initiation stage, oxirane is coordinated onto the surface Zn sites bonded with CA molecules, with subsequent nucleophilic attack of a weakly coordinating ligand (initiator, P123, H_2_O) or dissociated Cl^−^ on the coordinated oxirane to generate propagating species, along with insertion of monomer unit and proceeds until monomer is consumed completely (path A in Figure 9). Initiation via coordinative route most properly proceeds on the surface of the catalyst, where additional coordinating ligands are mainly located. Alternatively, the monomers adsorbed at internal Zn^2+^ sites undergo an activated-chain end (ACE) mechanism to afford a carbocation intermediate, followed by iterative addition of a monomer until the growing species are exposed to external ligands (path B). In the absence of an initiator, the reaction may be proceeded by both ACE and coordinative mechanism until cationic growing species are terminated completely, resulting in the formation of high-MW fractions and high PDI values. In the presence of an initiator, chain propagation via ACE is suppressed.

The effect of CA, which is a part of DMCCs, is directly related to the type and energy of Zn–O coordination: in highly active catalysts, the interaction between CA molecules and Zn^2+^ sites must be neither too weak (providing substitution of H_2_O during preparation of the catalyst) nor too strong (providing substitution by oxirane monomer at the initial stage of polymerization). Therefore, the dormant Zn^2+^ sites formed by the adsorption of γ-diketones or β-ketoesters (C=O^…^Zn coordination) exhibited much higher activity than those in enol form of β-diketones (C–O–Zn bonding) [52].

In the frameworks of conventional concepts of the nature of catalytic species, single electrophilic Zn ions in different ligand environment represent catalytic sites. However, in many works, devoted to the coordination–insertion mechanism of ring-opening polymerization, the role of bimetallic catalyst species was proposed and proven [109,110,111,112,113]. Based on DFT modeling results for Zn_2_[Co(CN)_6_]Cl [52], the distance between Zn atoms (4.4 Å) meets the possibility of bimetallic coordination–insertion mechanism for Zn/Co DMCCs. This concept was further developed in theoretical studies of PO/CO_2_ copolymerization (see below).

**Figure 10:**
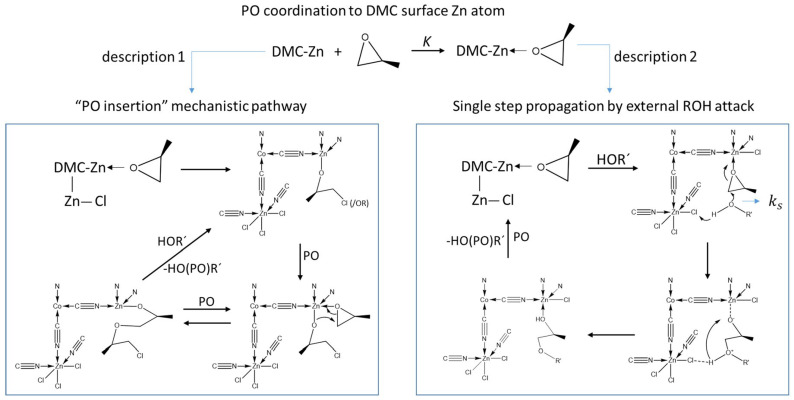
Competing mechanistic descriptions of the DMC-mediated propoxylation of HOR, insertion polymerization (**left**) and stepwise addition by external nucleophilic attack (**right**). Reprinted with permission from [104]. Copyright (2024) Royal Society of Chemistry, London, UK.

The possibility of alternative reaction mechanisms (Figure 10) meeting the kinetics of DMC-catalyzed polymerization of PO was studied and discussed in [104]. Direct experimental evidence in favor of binuclear mechanism (coordination of PO molecule and its reaction with an external polymer chain end) were based on kinetic measurements; therefore, the catalytic action of Zn/Co DMCCs can be more readily understood by considering them as an individual “microreactors”, which brings to the fore the factors of the PO diffusion rate and poly(PO) segment length.

DFT modeling of the mechanism of PO polymerization using DMCCs was presented and discussed in a few works. In 2015, the cluster model of Zn/Co DMC complex Zn_2_[Co(CN)_6_]X (X = Cl, OH, OEt, O*^t^*Bu) was used for the further theoretical study of the ROP of oxirane as a model substrate [99]. The calculations (PBE functional), performed with the use of Zn–OH sites, showed the preference of the ROP on Zn sites at (001) facets. Revealing the influence of the number of O–Zn bonds (three for (100) facets and two for (001) facets) is important for further understanding of the catalytic behavior of Zn/Co DMCCs. However, the consideration of Zn–OH (or Zn–OR) species as main centers of oxirane’s polymerization seems doubtful in our opinion.

**Figure 11:**
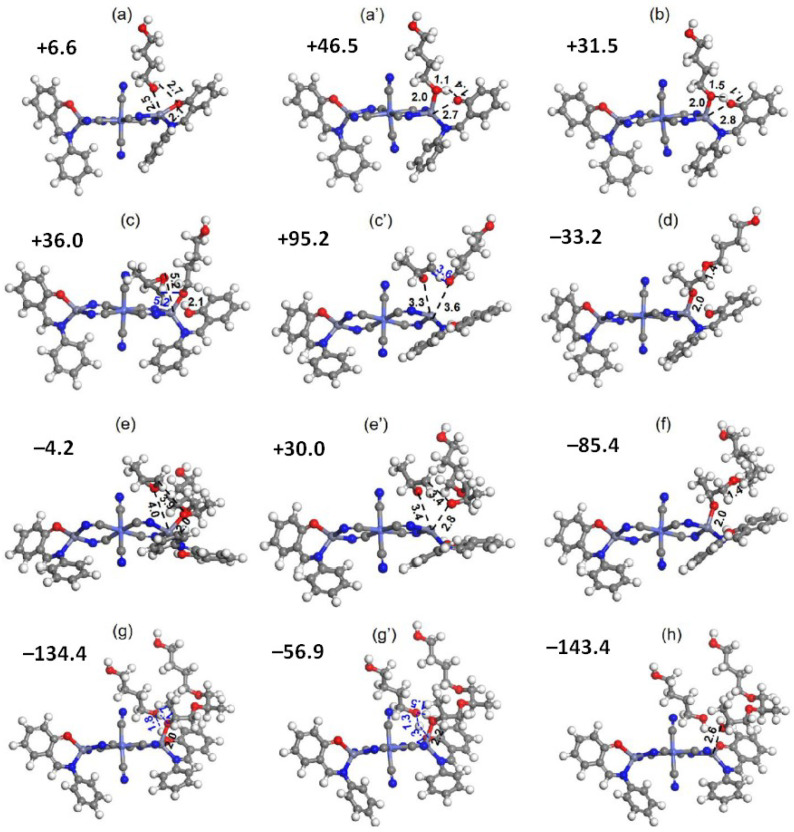
Geometries of intermediate and transition states formed during the ROP of PO reaction over DMC-4 catalyst. Color code: Co (light blue), Zn (ash color), N (blue), C (black), O (red), and H (white). Free energies relative to starting reagents (DMC complex and butane-1,4-diol) are also given (kJ∙mol^−1^). Reprinted with permission from [114]. Copyright (2023) Elsevier B. V., Amsterdam, The Netherlands.

DFT modeling of the ROP of PO, catalyzed by Zn/Co DMCCs, was also carried out recently by Verma et al. on a molecular level (PW91 functional, double numerical plus polarization basis set) as a part of the study of complexes, prepared with the use of Schiff bases as CAs [114]. The structures of the key intermediates and transition states for HO(CH_2_)_4_OH-initiated ROP of PO, catalyzed by the complex of (*E*)-2-((phenylimino)methyl)phenol, are presented in Figure 11. The authors proposed a single-site reaction coordination–insertion mechanism for this process; the overall calculated activation barrier for the polymerization of PO was +95.2 kJ∙mol^−1^ and the overall reaction was exergonic by −78.2 kJ∙mol^−1^.

The aspects of polymerization kinetics are crucial to industrial production of polyols. As applied to DMC-catalyzed polymerization of oxiranes, a kinetic model was proposed recently by Jupke et al. [115,116]. This model covers the characteristic “catch-up” kinetics (when DMCCs prefer to grow short chain polyols while long chain polyols stay unreacted), favoring the growth of relatively small macromolecules, and undesirable high molecular weight tailing. This model meets experimental results, observed in (semi-)batch operations during production of PO-based polyols, and was used in recent development of the process of polyol production by the use of a semi-batch reactor with external loop for continuous PO feed [117].

### 3.2. DMC Catalysts in Copolymerization of Oxiranes with CO_2_

The mechanism of copolymerization of PO and CO_2_ is not yet clear. The microstructure of copolymers clearly indicates the absence of anhydride fragments, since insertion of CO_2_ at Zn–OC(O)R is thermodynamically forbidden [118]. The growth of a copolymer chain includes subsequent coordination and insertion of oxirane and CO_2_ molecules. The degree of introduction of CO_2_ *F*_CO2_, determined as a fraction of carbonate fragments relative to the total number of ether and carbonate units, is an important characteristic of PEC; for polycarbonates (PC) *F*_CO2_ = 100%.

Most researchers agree that PO/CO_2_ copolymerization proceeds on single metal center (Zn atom for Zn/Co DMCCs) and is influenced by ligand environment of the Zr^2+^, reaction media and conditions [6]. Important mechanistic patterns were revealed when studying Zn/Co DMC-catalyzed copolymerization of cyclohexene oxide and CO_2_ [119]. The authors showed that Zn–OH fragment is the real active site for initiating the copolymerization. Importantly, the introducing of *^t^*BuOH into the reaction mixture suppressed the formation of the oligoether fragments, ultimately leading to alternating polymer (*F*_CO2_ = 97%).

**Figure 12:**
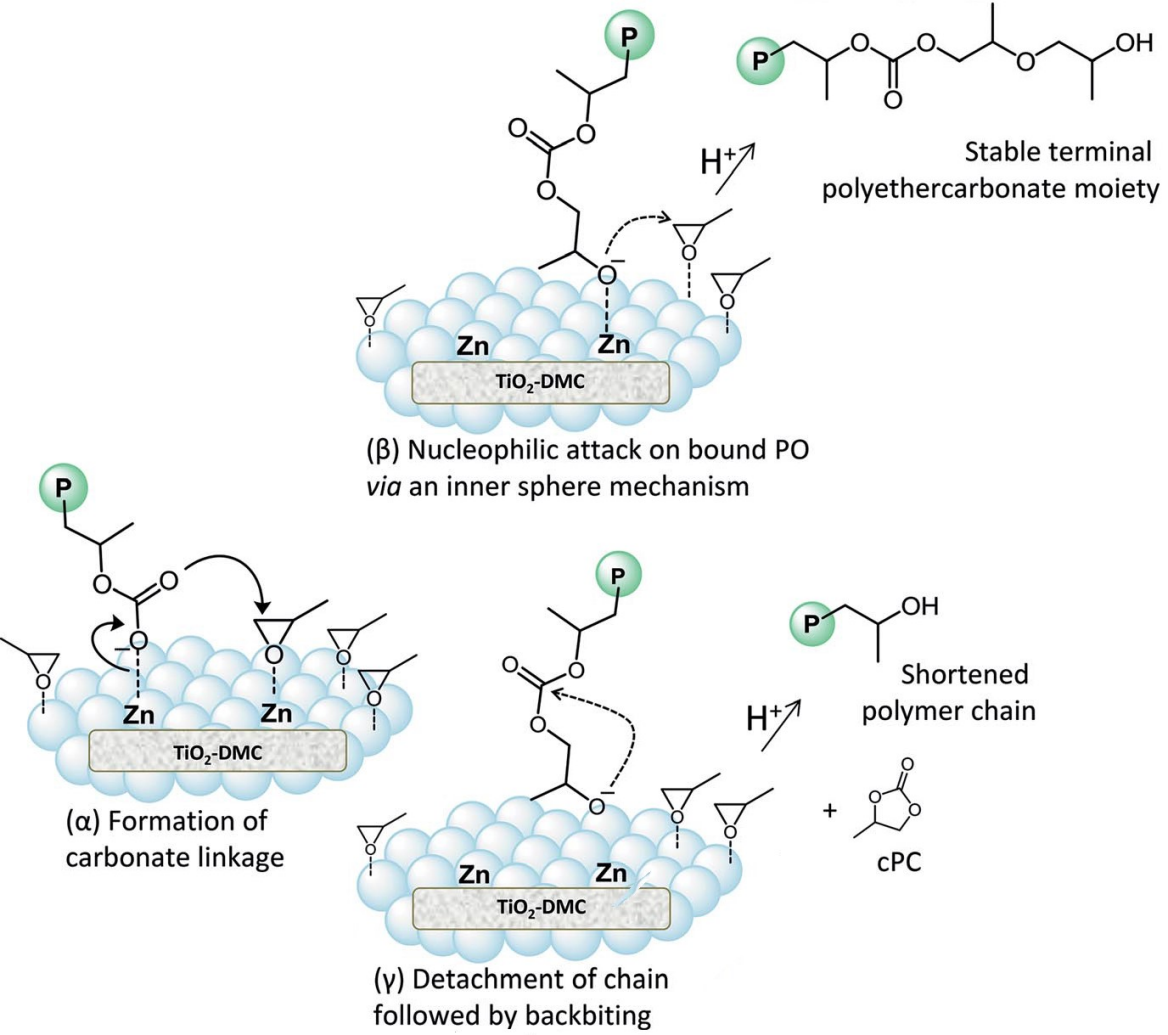
Mechanism suggested for the formation of stable poly(ether-carbonate)s with a high content of carbonate linkages in repeating units with a non-alternating sequence of comonomers. Reprinted with permission from [75]. Copyright (2016) Wiley-VCH Verlag GmbH & Co., Weinheim, Germany.

DMC catalysts with enhanced Lewis acidity (e.g., TiO_2_-supported) demonstrate high selectivity of copolymerization with low formation of cyclic carbonates. According to the authors of [75], Lewis acidic character of the TiO_2_ support leads to stronger coordination of the PO molecules at the Zn centers or provides additional coordination sites for the PO molecules in the vicinity of the Zn centers. A higher concentration of PO on the surface will enhance the probability of transferring the terminal carboxylate group (pathway α → β in Figure 12) or terminal RO^−^ group to a neighboring PO molecule (β). On the contrary, the probability of dissociation of a terminal group, followed by nucleophilic attack of the alcoholate end group at a vicinal carbonate group with a formation of cyclic carbonate, is reduced (γ in Figure 12).

**Figure 13:**
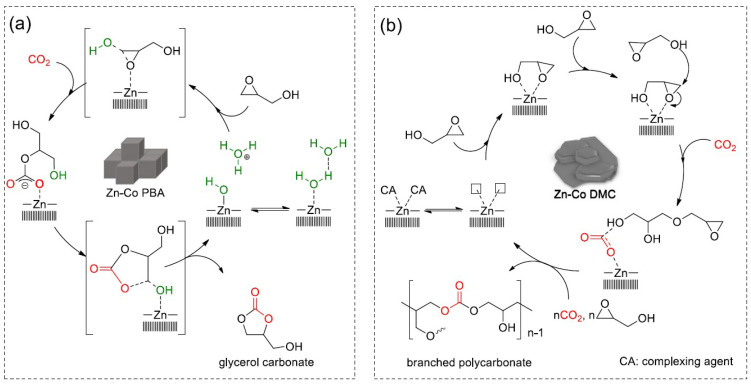
Proposed mechanism for the Zn/Co PBA-catalyzed cycloaddition of CO_2_ to glycidol (**a**) and Zn/Co DMC-catalyzed copolymerization of CO_2_ and glycidol (**b**). Reprinted with permission from [66]. Copyright (2023) Elsevier B. V., Amsterdam, The Netherlands.

The difference in catalytic behavior between well-defined Prussian blue analog Zn_3_[Co(CN)_6_]_2_∙5H_2_O and amorphous Zn/Co DMC complex, prepared using Me_3_SiOH and P-123 as CAs, was clearly demonstrated by Kim et al. by the example of the reaction of glycidol with CO_2_: in the former case, cyclic carbonate was formed, and in the latter case, copolymerization was observed [66]. Formation of glycerol carbonate was explained using DFT modeling (PBE generalized gradient approximation functional, double numerical basis set with polarization) on the assumption of the participation of surface Zn–OH species in the catalytic cycle; the calculated activation barrier for this reaction (Figure 13a) amounted to ~10 kcal∙mol^−1^ (G scale). More acidic and less sterically hindered Zn sites in amorphous Zn/Co DMCC initiate polymerization (Figure 13b).

**Figure 14:**
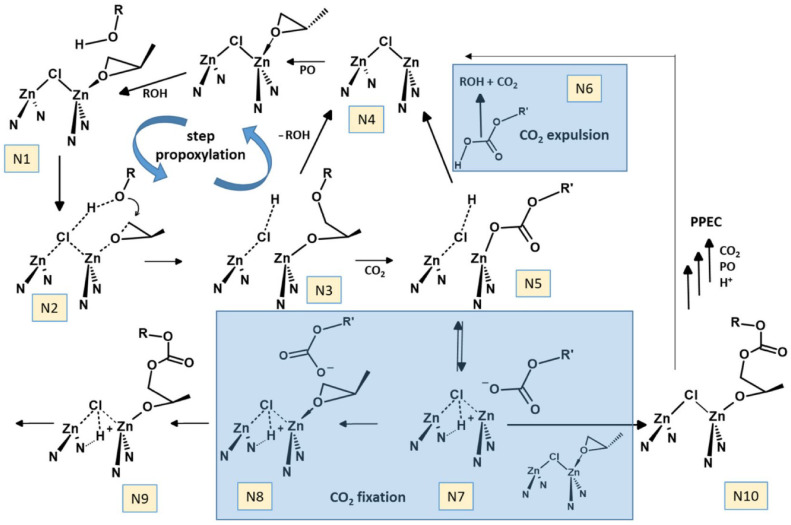
Propoxylation in combination with carboxylation mediated by “non-basic” DMCCs, i.e., those with few basic entities. Reprinted with permission from [120]. Copyright (2020) MDPI.

**Figure 15:**
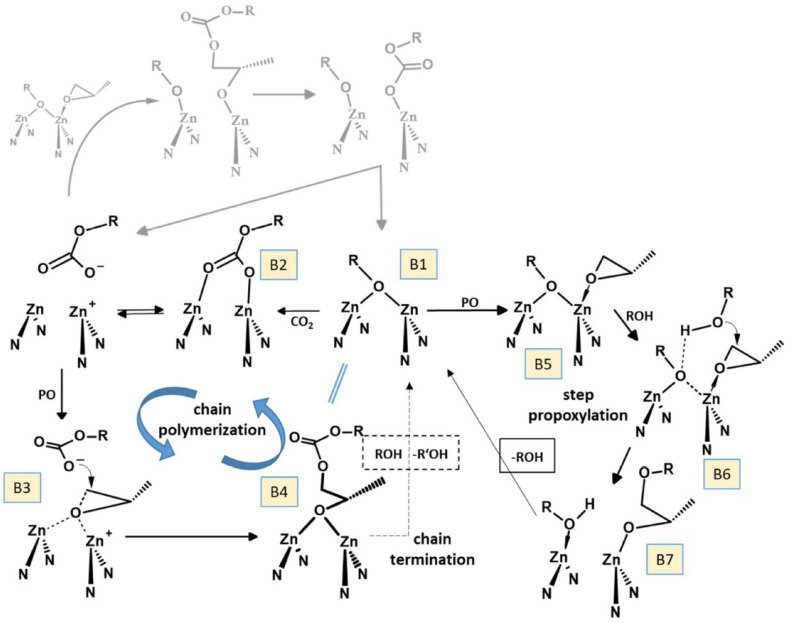
PPEC formation by DMC with anionic sites (alternative CO_2_ fixation pathway in grey). Reprinted with permission from [120]. Copyright (2020) MDPI.

It also should be noted here that the concept of binuclear mechanism of the action of Zn/Co DMC catalysts was also applied to PO/CO_2_ copolymerization. Based on results of previous studies [99], Stahl and Luinstra proposed two bimetallic reaction pathways, distinguishing by the nature of catalytic sites (non-basic and basic, see Figure 14) [120] and including only low activation energy transformations. The reaction sequence may be formulated in form of an external attack of a hydroxyl chain end, proton transfer, and liberation of a coordination site for PO activation (Figure 14, N1–N4) with assistance of Cl ligand (N3). The incorporation of CO_2_ occurs via insertion into a Zn–OR bond (N5). Subsequent protonation would lead to unstable alkyl carbonic acid (N6) that decarboxylates (non-productive cycle). Interruption of this cycle is achieved when the intermediate carboxylate is not protonated but reacts with activated PO to regenerate Zn–OR (Figure 14, N7–9 or N7, N10). This catalytic scheme was proposed for DMC-Cl systems with zero response of PEC composition on CO_2_ concentration in the feed. When using DMC-NO_2_ catalysts, prepared during studies, the CO_2_ incorporation was positive dependent on CO_2_ concentration, which found explanation in the frameworks of the mechanism presented in Figure 15. After exchange with hydroxyl compounds in the solution, anionic chain ends (B1), formed on the surface, may insert CO_2_; the CO_2_ insertion rate becomes dependent on the CO_2_ concentration in the feed, and this process competes with propoxylation (B5–B7).

### 3.3. Mechanism of Transesterification of Cyclic Carbonates

**Scheme 6:**
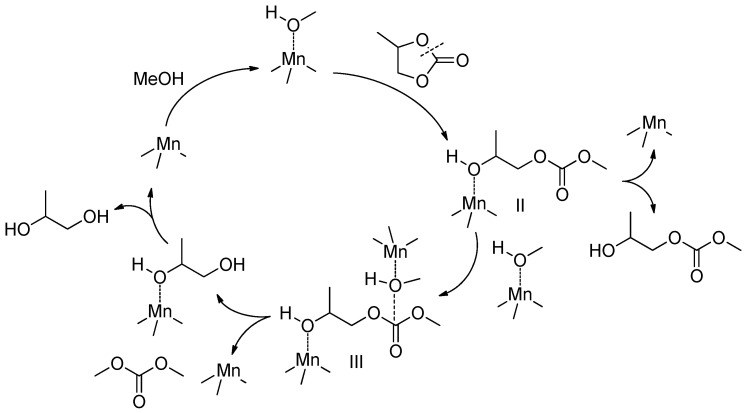
The proposed mechanism of methanolysis of propylene carbonate, catalyzed by Mn/Fe DMCC [121].

Transesterification of cyclic carbonates (see Section 4.6) is not the most important field of DMCC application; however, the mechanism of this reaction deserves a mention in view of the bimetallic concept mentioned earlier. As was shown in [121], methanolysis of propylene carbonate, catalyzed by Mn/Fe DMC complex, can be described by the following mechanistic steps (Scheme 6): (1) activation of MeOH by electrophilic Mn center, (2) interaction of propylene carbonate with activated MeOH to form intermediate II, (3) reaction of the intermediate II with activated MeOH (second Mn atom) to form intermediate III, (4) decomposition of intermediate III with a formation of (MeO)_2_C=O and propylene glycol, completing the catalytic cycle.

## 4. Actual Processes and Products

### 4.1. Preparation of Polyols

**Scheme 7:**
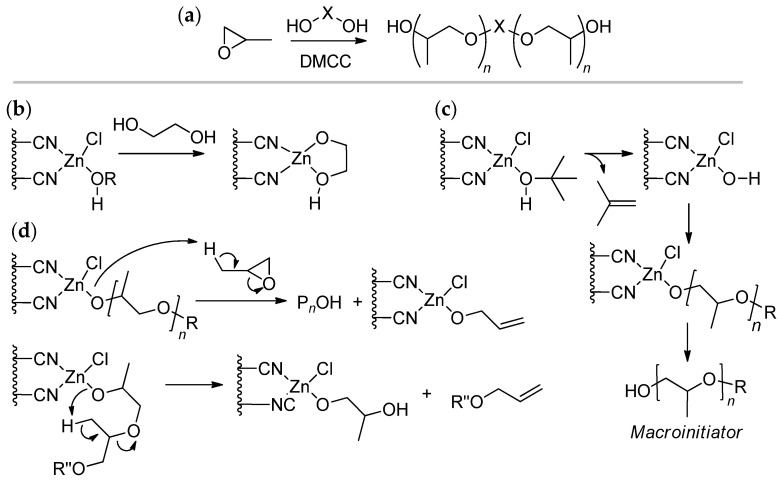
Synthesis of polyols, catalyzed by DMCCs (**a**), limitations and concerns of this process: deactivation of the catalyst (**b**), formation of high-MW fraction (**c**) and unsaturations (**d**).

The main industrial application of DMCCs is their use in production of high molecular weight polyalkylene ether polyols; propylene oxide is the most used monomer (Scheme 7a) [53].

Since 1966 [17], polymerization of PO and other substituted oxiranes has been intensively studied and used for the production of high-quality polyols that are highly in demand in the production of polyurethanes [20,122,123,124]. In 2005, Ionescu completed a detailed review of the use of DMCCs in the synthesis of polyols [125]. However, in the years since, significant progress in this area has been made, and catalyst concentrations of as little as 15–50 ppm are sufficient for industrial production of polyols [7].

#### 4.1.1. Zn/Co DMCCs in the Synthesis of Polyols

Preparation of polyols using Zn/Co DMCCs is complicated by the impossibility of using low-MW initiators such as propylene glycol and glycerol. It is assumed that 1,2-diols and glycerol form stable chelate complexes, thereby inhibiting coordination of monomers [24] (Scheme 7b). As a consequence, polymerization of PO with the use of Zn/Co DMCCs is usually initiated by oligomeric polyols, prepared by KOH-catalyzed condensation of PO [10]. Besides these polyols, low-MW paraformaldehyde [126], castor oil [127], oxalic acid [128], 1,3,5-benzenetricarboxylic acid [129], and 1,2,4,5-benzenetetracarboxylic acid [130] were used as initiators. Note that Zn/Co DMCC, prepared by the reaction of H_3_[Co(CN)_6_] with Zn(II) 2-ethylhexanoate, was active in polymerization of PO, initiated by propane-1,2-diol [61]. In 2024, Verma et al. reported the results of kinetic experiments of PO polymerization, catalyzed by Zn/Co DMC complex, prepared with the use of EDTA as a CA; for this type of catalyst, ethylene glycol showed no chelating effect and acted as an initiator and chain transfer agent [131].

Another critical concern in DMC-catalyzed polyol production is the formation of high-MW impurities [99,117,120,132] and unsaturations [53], attributed to action of Zn–OH initiators, initially presented in DMCC or formed as a result of dehydration of *^t^*BuOH (Scheme 7c) and conversion of methyloxirane to allyl alcohol or termination of the polyether chain with a formation of PPO containing allyl end-group (Scheme 7d).

Polymerization of PO, catalyzed by Zn/Co DMCCs, is usually conducted in bulk at elevated temperatures (e.g., 120 °C, polymerization time 4–8 h [133]); ^13^C NMR analysis of poly(propylene oxide) (PPO) showed high regioselectivity of polymerization (head-to tail sequence). PPO, obtained with the use of Zn/Co DMCCs, had narrow MWD and low content of unsaturations. In early works [38], studies of the influence of DMCCs composition and morphology on their characteristics also revealed that the amorphous catalyst prepared from ZnCl_2_ and *^t^*BuOH had the highest activity. The addition of minimal amounts of CaCl_2_ to DMC formulation increased its catalytic activity [107], but the reasons for this phenomenon have not been explained.

The synthesis of Zn/Co DMCCs with the use of *^t^*BuOH as a CA requires high consumption of *^t^*BuOH. The use of lactates in combination with polyols results in catalysts that allow obtaining of high-quality polymers (*M*_n_ = 4.1–4.9 kDa, *Ð*_m_ = 1.08–1.25) with unsaturation lower than 0.0034 meq∙g^−1^ [107]. Replacement of *^t^*BuOH by EDTA at the stage of preparation of Zn/Co DMCC resulted in obtaining of a catalyst with similar morphology, but low TOF = 9625 h^−1^ and unsaturation level of 0.009 meq∙g^−1^ [67]. Promising results in the development of efficient catalysts for the synthesis of polyols were obtained recently by Verma et al.: when using Schiff bases instead of *^t^*BuOH with PEG as a co-CA, obtained Zn/Co DMCCs showed very high catalytic activities with a short induction period, outperforming conventional *^t^*BuOH-based catalysts on these parameters [114]. The use of cyclic carbonates instead of *^t^*BuOH at the stage of the preparation of Zn/Co DMCCs resulted in a twofold increase of TOF from 212 to 467 min^−1^ (115 °C, 85 ppm of the catalyst) even though much lower amounts of CA and ZnCl_2_ were used during preparation of the catalyst [58]. A similar increase of the catalytic activity was detected for Zn/Co DMCCs, prepared with the use of dialkyl phosphites or trialkyl phosphates as Cas; e.g., (EtO)_2_P(O)H-based catalyst showed TOF = 725 min^−1^ at 115 °C [59].

Relatively recently [62], a number of well-defined Zn/Co DMCCs were tested in comparison with the benchmark DMC (Zn/Co ratio ~2:1) currently used in the industry. The experiments were conducted with a continuous feed of PO, making it difficult to compare inherent activities of the catalyst’ samples; however, comparison with benchmark DMC allows estimation of this parameter. As can be seen in Table 1, even in the absence of Cl in its composition (Table 1, Entry 12), the catalyst of the formula DMC-(OAc/OtBu)(0.95/0.08) displayed the yield surpassing that of the benchmark DMC. Following closely were the DMC-(OAc/OtBu/Cl) series (29–31 g yields) and DMC-Cl (28 g yield). The initiation time was quite short (several minutes) in most cases, except [DMC-Cl][MeOCMe_2_OH], DMC-OtBu, and DMC-(OAc/OtBu)(0.31/0.71), which required external heating for initiation. The polyols generated with DMC-Cl exhibited high unsaturation levels (0.022–0.024 meq∙g^−1^), whereas those produced by DMC-OCMe_2_OMe and DMC-OtBu showed very low unsaturation levels, matching that of the benchmark DMC (0.004 meq∙g^−1^). Zn/Co DMC complexes, prepared with the use of different dicarbonyl compounds, showed broad spectrum of catalytic properties; the highest productivity (TOF = 569 min^−1^ at 130 °C) was demonstrated by the complex prepared using diethyl malonate as a CA, and the obtained polymer had *M*_n_ = 2.9 kDa, *Ð*_m_ = 1.02 and unsaturation of 0.0017 meq∙g^−1^ [57].

To increase catalyst activity, stability and reusability, supported Zn/Co DMCCs were prepared in recent years. In particular, MCM-41@Zn/Co DMCC (0.05 wt%) catalyzed polymerization of PO with 90.6% conversion, not inferior to conventional DMCCs, but showed good reusability (at least 7 cycles) [72]. To access high-MW polyols, Kim et al. proposed a sequential approach, based on conducting of a series of successive stages, affording PEs with *M*_n_ up to 43.6 kDa [134].

Besides PO, other oxiranes were studied in homopolymerizations catalyzed by Zn/Co DMC complexes. For example, epichlorohydrin was polymerized using tri-(2-hydroxyethyl) isocyanurate as an initiator, and the reactivity of the monomer was comparable with reactivity of PO [135]. Regioselective ROP of enantiomerically pure epichlorohydrin on Zn/Co DMCC was not accompanied by epimerization; a solid polymer was obtained in contrast with the liquid product of the homopolymerization of *rac*-epichlorohydrin (*T*_g_ = −38 °C) [136].

Preparation of hyperbranched polyols via copolymerization of PO and glycidol was studied by Gu and Dong [137]. During later studies, Kim et al. showed that Zn/Co DMC complexes in combination with diethyl malonate or ethyl acetoacetate efficiently catalyze homopolymerization of glycidol without the use of an initiator; degrees of branching (DB) were determined by the type of monomer addition method: DB = 0.25–0.27 for solvent-free batch process and DB = 0.51–0.57 for semi-batch polymerization [138]. To incorporate polar structural units to poly(PO), Frey et al. proposed the use of glycidyl methyl ether (GME) as a comonomer; commercial DMC catalyst from BASF was applied in their studies [139]. Copolymers with *M*_n_ = 1.9–4.5 kDa, *Ð*_M_ < 1.29 and up to 45 mol% GME content were obtained. It is noteworthy that copolymers had a random microstructure (r_PO_ = 1.40 ± 0.01, r_GME_ = 0.71 ± 0.01), which qualitatively distinguishes GME from other substituted oxiranes.

In 2024, a zeolite-supported layered Zn/Co DMC was used as a catalyst for polymerization of butylene oxide [76]. The high catalyst’s performance was attributed to the high dispersion of DMC and the presence of additional Lewis acid sites on the support surface. Products with *M*_n_ = 1.4–4.5 kDa, *Ð*_M_ = 1.08–1.53 were obtained. Poly(butylene oxides) were characterized by viscosity index of up to 242 and relatively low pour point values (as low as −45 °C) which made it possible to consider these materials as an additives to mineral lubricating oils.

#### 4.1.2. Other DMCCs in the Synthesis of Polyols

As was shown in [79], polymerization of PO in the presence of Zn/Fe DMCCs, prepared using Na_2_EDTA as a CA, can be initiated by ethylene glycol. Under optimized conditions (350 ppm of the catalyst, 105 °C, 5 bar, 24 h), PPO with *Ð*_M_ = 1.29 and OH value of 4.9 mg_KOH_∙g^−1^ was obtained; during optimization, a wide spectrum of polymers (*M*_w_ = 0.16–19.67 kDa, *Ð*_M_ = 1.05–3.01) were synthesized.

Zn/Ni DMCCs, prepared in the absence of CA by subsequent reaction of NiCl_2_ with KCN and Zn(OAc)_2_ ([NiCl_2_]/[KCN]/[Zn(OAc)_2_] = 1:6:6), demonstrated high catalytic activity in polymerization of PO and other oxiranes, but were inferior to Zn/Co DMCCs in activity [89]. Co/M DMCCs, synthesized from CoCl_2_ and K_2_[Ni(CN)_4_], K_4_[Fe(CN)_6_], K_3_[Fe(CN)_6_], or K_3_[Co(CN)_6_] in the presence of *^t^*BuOH and PTMEG, were less active in comparison with the Zn/Co DMC complexes; only Co/Ni DMCC approached the benchmark catalyst in productivity [85].

#### 4.1.3. Post-Functionalization of PEs

**Scheme 8:**
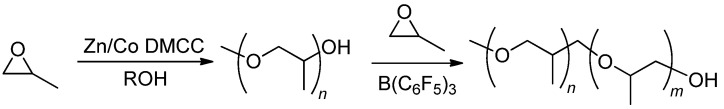
Synthesis of high primary hydroxyl polyetherols via sequential catalysis [141].

Low-MW polyols, obtained by ROP of PO, are usually intended for the production of polyurethanes. However, these materials, obtained using DMCCs, contain a low degree of primary hydroxyl groups having higher reactivity at the stage of the reaction with isocyanates. The commonly use procedure of the introduction of –CH_2_OH fragments to PE microstructure is a base-catalyzed ethylene oxide capping [140]. To simplify the preparation of –CH_2_OH-terminated polyols, Raghuraman et al. proposed the use of tris(pentafluorophenyl)borane at the final stage of ROP of PO (Scheme 8) and co-ROP of PO and butylene oxide; the content of more than 65% of primary hydroxyl end-groups was achieved [141], and the loading of ~150 ppm B(C_6_F_5_)_3_ turned out to be sufficient.

### 4.2. Copolymerization of Oxiranes with CO_2_ and Other C1 Comonomers

**Scheme 9:**
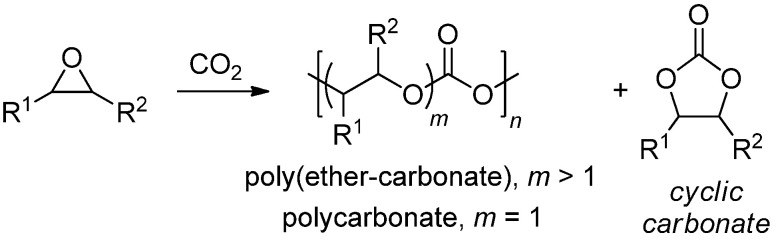
Copolymerization of oxiranes with CO_2_.

Copolymerization of oxiranes with CO_2_ may result in the formation of poly(ether-carbonates) and polycarbonates (PEC and PC, respectively); the latter copolymers represent macromolecules with alternating sequence of comonomer units [8,15] (Scheme 9). It is believed that DMCCs usually catalyze formation of PEC; however, in some cases, copolymers, obtained in the presence of DMCCs, are approaching the composition of PCs. The products of DMCC-catalyzed copolymerization of PO and CO_2_ represent polyols with carbonate inserts that are also of interest to polyurethane production [142]. Cyclic carbonates are side products of this reaction. Given that cyclic carbonates are demanded fine chemicals, this process appears to be waste-free and environmentally friendly, which explains continued interest of the researchers to DMC-catalyzed copolymerization of oxiranes and CO_2_. *F*_CO2_ is an essential property of PO/CO_2_ copolymers: as was shown in [143], polyols with high carbonate content provide higher thermo-oxidative stability of polyurethanes with reduced generation of volatile organic compounds (e.g., acetaldehyde) during decomposition. Another substantial factor is a structure of initiator/chain transfer agent/chain extender used during preparation of PO/CO_2_ copolymers [144]; in this particular case, fine tuning of these chemicals allowed to select copolymer suitable for the production of polyurethanes compatible with anti-corrosive polyanilines.

PO is a most commonly used monomer, and splitting on subsections below was made in view of this fact. The products of copolymerization of other oxiranes with CO_2_ turned out to be less attractive for their use as a material of the new millennium with a few exceptions (see below). Other C1 comonomers (COS, CS_2_) can also react with oxiranes in the presence of DMCCs, and these processes will also be considered in this Section.

#### 4.2.1. Zn/Co DMCCs in Copolymerization of Propylene Oxide and CO_2_

In comparison with ROP of PO, copolymerization of PO with CO_2_ proceeds much more slowly. The process is limited by the same factors as the constraints and challenges of PO homopolymerization except formation of high-MW polymer fraction. In early study of Gao et al. [145], PECs with *M*_n_ = 1.8–6.4 kDa, *Ð*_M_ = 1.14–1.83 and *F*_CO2_ = 15.3–62.5% were obtained, and productivity of Zn/Co DMCC (prepared by standard method from K_3_[Co(CN)_6_], ZnCl_2_ and *^t^*BuOH) was up to 10 kg∙g_cat_^−1^. The formation of propylene carbonate was not more than 8%. PO oligomers were used as initiators in these experiments. Zn/Co DMCCs, prepared using ZnX_2_ (X = F, Cl, Br, I), *^t^*BuOH, and poly(tetramethylene ether glycol), were studied in PO/CO_2_ copolymerization [27]. ZnF_2_-based catalyst showed negligible activity, whereas other ZnX_2_-based catalysts demonstrated comparable productivities, ~510 g∙g_cat_^−1^ (50 °C, 9.6 bar CO_2_, 24 h), *F*_CO2_ were 22, 36, and 32% for X = Cl, Br, and I, respectively. When using polyvinylpyrrolidone as a co-CA during preparation of Zn/Co DMCC with standard co-precipitation in the presence of *^t^*BuOH, the modified catalyst exhibited higher catalytic activity than the conventional DMCCs, with a nearly 10% increase in PO conversion. The most critical breakthrough was a reduction of the formation of propylene carbonate to ~5 wt% [146]. It was also shown that *^t^*BuOH can be substituted by pluronic P-123 at the stage of preparation of Zn/Co DMCCs, but careful calcination is critical to providing reduced crystallinity and enhanced formation of the active monoclinic phase [147].

Conventional Zn/Co DMCCs usually provide moderate carbonate content (up to 40 mol%), but the use of [ZnCl]_2_[HCo(CN)_6_]∙MeOH resulted in copolymers with *F*_CO2_ ~ 60% [44], introducing of 1,10-decanediol as chain transfer agent provided the formation of low-MW PECs (*M*_n_ ~ 2 kDa) suitable for polyurethane production. The later study of Zn/Co DMCC-catalyzed PO/CO_2_ copolymerization revealed the preference of the formation of a copolymer containing 3–4 ether units, thereby confirming the mechanism of PO/CO_2_ copolymerization initiated by moderately acidic Lewis centers like Zn in Zn/Co catalysts [148].

Besides PO-based polyols, bisphenol A [149], 1,1,1-tris(4-hydroxyphenyl)ethane [150], oxalic acid [128], sebacic acid [151], low-MW paraformaldehyde [126], and polycarboxylic aromatic acids [129,130,152] were used as initiators. It was also shown that dicarboxylic acids may act as a chain transfer agents, thereby controlling molecular weight characteristics of PEC [151]. The nature of the initiator allows it to affect the architecture of the macromolecules (the number of –OH end groups), which is essential for the further use of PEC in polyurethane production.

A comparative study of a number of well-defined Zn/Co DMCCs and industrial DMC [62] was conducted with continuous feed of PO and CO_2_. As can be seen in Table 2 in comparison with Table 1 (PO homopolymerization), the catalytic activity decreased significantly under CO_2_ pressure, and a higher amount of catalyst was needed (11 vs. 4.0 μmol-Co) with a lower PO feed rate (0.50 vs. 1.25 mL∙min^−1^). The catalysts containing (tBuO)Zn^+^ or (AcO)Zn^+^ units, including the benchmark DMC, showed high activities, yielding 18–28 g of polyols. A dramatic productivity difference was observed between DMC-OAc and the structurally similar Zn_2_[Co(CN)6](OAc)·4H_2_O (19 vs. 6.8 g, respectively). DMC-(OAc/OtBu)(0.95/0.08) and DMC-(OAc/OtBu)(0.68/0.31) exhibited the highest yield (26–28 g) with short initiation time (15 min), proving superior to the benchmark DMC (25 g yield; initiation time 36 min). In terms of the dispersity of the resulting polymer, DMC-(OAc/OtBu)(0.95/0.08) also demonstrated a fairly narrow unimodal GPC curve with the lowest *Ð*_M_ = 1.43. Formation of propylene carbonate is a critical concern in PO/CO_2_ copolymerization. Catalysts with poor activities, such as DMC-Cl, DMC-OCMe_2_OMe, and DMC-(OCMe_2_OMe/Cl)(0.5/0.5) initiated significant cyclic carbonate formation (9.2–25 mol%). The catalysts with good activities produced small amounts of cyclic carbonate (<5 mol%). Among these, DMC-OtBu, DMC-(OAc/OtBu/Cl)(0.59/0.38/0.15), and DMC-(OAc/OtBu/Cl)(0.74/0.24/0.10) performed with carbonate formation below 2 wt%. It is very interesting and important that low unsaturation levels in PO polymerization correlate with low cyclic carbonate formation in PO/CO_2_ copolymerization. Both side processes can be catalyzed by homogeneous Zn species [153]; therefore, the possibility of leaching of such homogeneous species could be minimized during preparation of the DMCCs.

Interesting results were obtained in PO/CO_2_ copolymerization, catalyzed by Zn/Co DMCC, prepared using ball milling, in the presence of γ-Al_2_O_3_ as a co-catalyst [154]. Polymerization proceeded with higher rates, and the thermal stability of copolymers was greatly improved, up to 379 °C. Another way of improving the yield and molecular weight characteristics of PECs was the use of hybrid catalyst comprising Zn/Co DMCC and Zn(II) glutharate in 1:10 molar ratio by Zn [155]; *M*_n_ up to 58 kDa, *F*_CO2_ = 97.7%, and productivity of 508 g∙g_cat_^−1^ were achieved.

Concluding this Section, it should be noted that the properties of copolymers, obtained with the use of Zn/Co DMCCs, may be largely affected by the type and quantity of the initiator used (in other words, relatively large amounts of specific initiators can be considered as a valuable components). For example, the use of bisphenol A as an initiator and chain transfer agent allows copolymers to be obtained, which can be used in further synthesis of polyurethanes with improved flame-retardant properties [149].

#### 4.2.2. Other DMCCs in Copolymerization of Propylene Oxide and CO_2_

Zn/Fe DMCCs, prepared by ball milling, were used in copolymerization of PO with CO_2_; however, high yields of cyclic carbonates were detected [80,81]. The higher CO_2_ incorporation during PO/CO_2_ copolymerization for Zn/Ni DMCCs in comparison with Zn/Co catalysts was revealed by Qi et al. in 2007 [28]. Nanosized Zn/Ni and Co/Ni DMCCs, synthesized by ball milling, in alternating copolymerization of PO with CO_2_ showed high catalytic activity; obtained after 24 h (40 bar, 60 °C), PEC had *M*_n_ up to 10.3 kDa, *Ð*_M_ = 1.45 and 83.5 mol% of carbonate linkages (Zn/Ni), whereas Co/Ni system catalyzed formation of PPC with *M*_n_ = 8.48 kDa, *Ð*_M_ = 1.44 and *F*_CO2_ = 73.7% [156]. Zn/Ni DMCC, prepared by co-precipitation using K_2_[Ni(CN)_4_], ZnCl_2_, and *^t^*BuOH, exhibited productivity ~500 g∙g_cat_^−1^, with *F*_CO2_ up to 60% [90].

Zn/Cr DMCC (1 wt%) at 70 °C and 40 bar catalyzed formation of PEC with *M*_n_ = 68.6 kDa and *Ð*_M_ = 1.68; *F*_CO2_ = 38.5% [93]. DMC complexes of the formula Co[M(CN)_4_] showed low (M = Pd, Pt) or moderate (M = Ni) activities in PO/CO_2_ copolymerization: at [PO]/[Co] ratio of 2530:1 at 130 °C and 54 bar of CO_2_ the maximum TOF was 1860 h^−1^; for copolymer with *M*_n_ = 74.3 kDa and *Ð*_M_ = 3.1, the *F*_CO2_ value was 20% [92]. Although the activity of Co[Ni(CN)_4_] was significantly lower than the Zn/Co DMCCs, the advantage of this system was the absence of propylene carbonate in the reaction products.

Hexane-1,6-diol initiated PO/CO_2_ copolymerization, catalyzed by Co/Ni DMCCs, under optimized conditions (120 °C, 43 bar of CO_2_) resulted in PECs (1.58 kg∙g_cat_^−1^∙h^−1^) with *M*_n_ = 2.6 kDa, *Ð*_M_ = 2.5, and *F*_CO2_ = 25% [91]. Ni_3_[Co(CN)_6_]_2_, Co_3_[Co(CN)_6_]_2_, Fe_3_[Co(CN)_6_]_2_, Ni_3_[Fe(CN)_6_]_2_, and Co_3_[Fe(CN)_6_]_2_ showed moderate activities in PO/CO_2_ copolymerization; the obtained PECs had *F*_CO2_ = 16–33%, *M*_w_ = 6–85.4 kDa, and *Ð*_M_ = 4.1–15.8 [86].

Relatively recently, hexacyanoferrate(II) complexes Ni_2_[Fe(CN)_6_], Co_2_[Fe(CN)_6_], KFe[Fe(CN)_6_], and Zn_2_[Fe(CN)_6_], synthesized by co-precipitation in the presence of *^t^*BuOH, were studied in PO/CO_2_ copolymerization (90 °C, 20 bar CO_2_, 24 h) [87]. PECs with *M*_w_ = 3.4–20.2 kDa, *Ð*_M_ = 4.0–5.4, and CO_2_ content of 9.3–18.1 wt% were obtained; the formation of propylene carbonate (1.4–19.8 wt%) was also detected. The TON values (in mol_PO_ per mol_M(II)_) were 84–223; under the same conditions, Zn/Fe DMCC demonstrated TON of 1279. The Table 3 gives a visual representation of catalytic performance of different DMC complexes in PO/CO_2_ copolymerization.

The complexes of the common formula M[Ni(CN)_4_] (M = Ni, Co, Fe, Mn) catalyzed PO/CO_2_ copolymerization yielding random PECs with *M*_n_ = 11.0–36.5 kDa, *Ð*_M_ = 2.5–5.0 and *F*_CO2_ = 13.1–42.4% [106]. Under optimized conditions, the Co/Ni DMCC provided the formation of copolymer and only 0.1 wt% of propylene carbonate. When compared to Zn/Co DMCC, the Co[Ni(CN)_4_], while exhibiting lower productivity and CO_2_ incorporation, outperformed the benchmark complex by the lack of induction time, higher copolymer MW, and selectivity of CO_2_ incorporation.

Among the latest publications on the subject, the work of Tebandeke et al. [158] also deserves attention: a composite catalyst, prepared from Zn(II) glutharate and Zn_3_[Cr(CN)_6_]_2_ complex in 15:1 ratio, showed low productivity (48 g∙g_cat_^−1^) but high *F*_CO2_ = 85.4%. This composite showed higher catalytic activity and selectivity compared to the individual components.

#### 4.2.3. Copolymerization of Other Oxiranes and CO_2_

Cyclohexene oxide (7-oxabicyclo [4.1.0]heptane) is a convenient substrate for the studies of catalytic copolymerization of disubstituted oxiranes with CO_2_. Already in the first study on the theme [26], TON of 3300 and *F*_CO2_ = 44% were achieved for Zn/Co DMCC, prepared with the use of *^t^*BuOH (90 °C, 36 bar, 2 h). The results of early studies in this field are described and discussed in our review [6], and productivities up to 8700 kg∙g_Zn_^−1^ and *F*_CO2_ up to 97% were observed when using optimized catalysts and conditions [119]. When using microwave irradiation, for Zn/Co DMCCs TOF = 25,177 h^−1^ was achieved [34]. Copolymers of cyclohexene oxide and CO_2_ represent amorphous materials, and the *T*_g_ value depends on *F*_CO2_; e.g., for copolymers with *M*_n_ ~ 5–10 kDa, *T*_g_ of 71.4, 81.4, and 98.6 °C corresponded to *F*_CO2_ = 35, 63, and 65%, respectively [50].

Copolymerization of glycidol and CO_2_ with a formation of highly branched polymers was efficiently catalyzed by amorphous Zn/Co DMCC, prepared by the reaction of ZnCl_2_ solution, containing trimethylsilanol, with K_3_[Fe(CN)_6_] solution, with subsequent treatment with aq. trimethylsilanol/P-123 [66].

Copolymerization of bulky oxiranes, e.g., styrene oxide, with CO_2_ is very sensitive to morphology of DMCCs. Nanolamellar Zn/Co DMCCs, prepared using *^t^*BuOH, THF, or MeOCH_2_CH_2_OH as CAs (~0.1 wt%) were studied in styrene oxide/CO_2_ copolymerization at 30–90 °C and 20–50 bar of CO_2_ [159]. In some experiments, highly regioselective copolymers with alternating degree > 99% were obtained; 2-benzyloxirane showed very similar behavior in copolymerization. Silica-supported Zn/Co DMCCs, prepared by sol–gel co-precipitation method [74], also showed high activity in copolymerization of styrene oxide with CO_2_, but the formation of cyclic carbonate was more pronounced (up to 17.4% when copolymer with ~1:1 comonomer ratio was obtained).

**Scheme 10:**
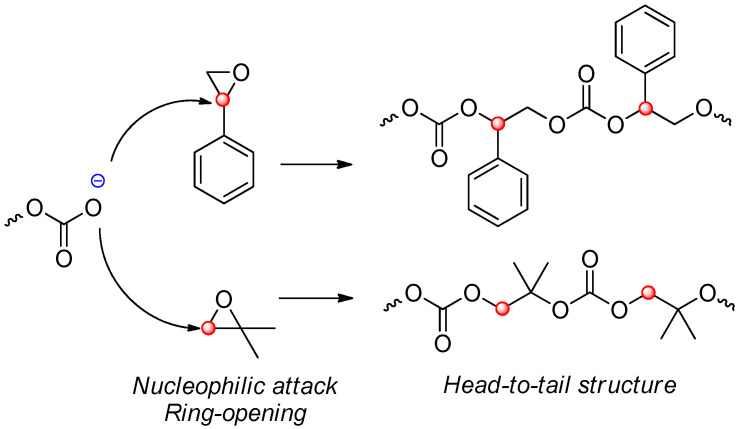
Proposed mechanism for regioselective styrene oxide/CO_2_ and isobutene oxide/CO_2_ copolymerization [160]. The red dot in the backbone represents the site proposed for attack by the carbonate anion.

**Figure 16:**
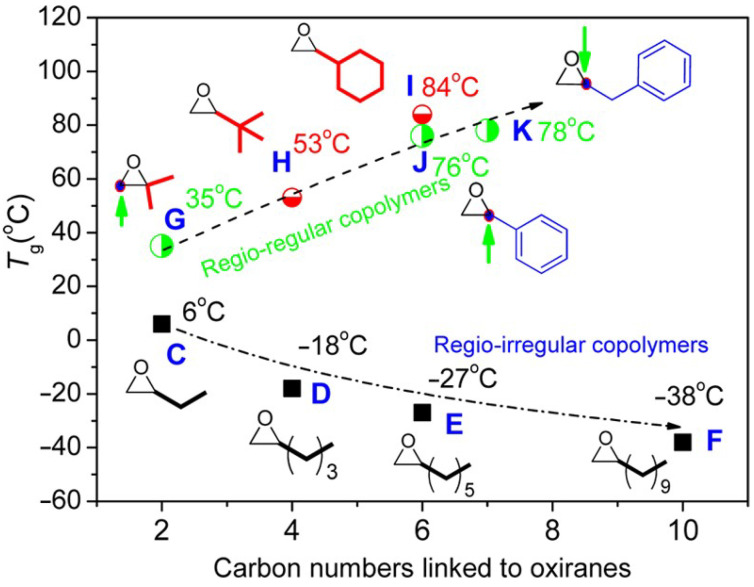
Substituent effect of the oxiranes on the glass transition temperatures (*T*_g_) of oxirane/CO_2_ copolymers (*F*_CO2_ = 91.5−99%; *M*_n_ = 6.6−93.2 kDa). Reprinted with permission from [160]. Copyright (2015) American Chemical Society, Washington, D.C., USA.

Comprehensive comparative study of the effect of substituent in oxirane on oxirane/CO_2_ copolymerization and properties of copolymer was conducted by Zhang et al. in 2015 for a sample of 11 oxiranes and Zn/Co DMCC [160]. Oxiranes with bulky substituents (2,2-Me_2_, *^t^*Bu, Cy, *n*-C_10_H_21_, Bn) formed copolymers with *F*_CO2_ up to 100%. The regioselectivity of oxirane/CO_2_ copolymerization was influenced by the electron induction effect: the electron-withdrawing substituents (Ph, Bn) induced regioselective ring-opening at the methine site, whereas for isobutene oxide/CO_2_ copolymerization the regioselective ring-opening occurred at the methylene site (Scheme 10); oxiranes with linear alkyl groups formed copolymers with lower regioselectivity. The glass transition temperatures (*T*_g_) of various oxirane/CO_2_ copolymers ranged from −38 to +84 °C (Figure 16) providing the use of similar copolymers as elastomers or plastics.

#### 4.2.4. Copolymerization of Oxiranes with Other C1 Monomers

**Scheme 11:**
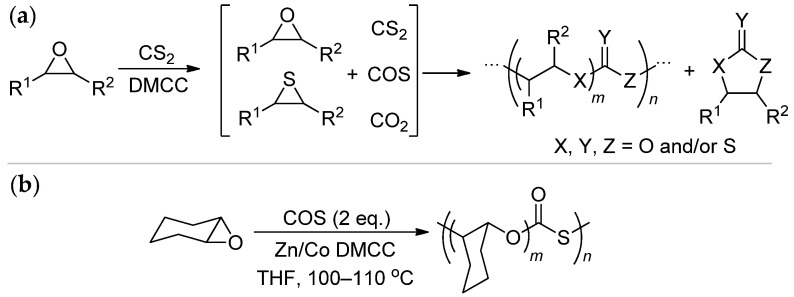
(**a**) O/S exchange reactions during copolymerization of CS_2_ and oxiranes [161]; (**b**) example of successful synthesis of well-defined sulfur-containing copolymer [162].

COS and CS_2_ are analogs of CO_2_, and the interest towards their using as C1 comonomers is fully justified. However, there are some sporadic publications in this field. The basic feature of copolymerization of oxiranes with COS or CS_2_ is O/S exchange side reaction with a formation of copolymers of rather complex composition and mixtures of cyclic thiocarbonates (which was observed in PO copolymerizations, catalyzed by Zn/Co DMCC [161]); under the action of DMCCs, CS_2_ and oxirane can transform to COS/CS_2_ and thiirane, respectively (Scheme 11a).

Nevertheless, in some cases scientists were able to synthesize well-defined copolymers. For example, cyclohexene oxide was copolymerized with COS in the presence of Zn/Co DMCC with a formation of poly(cyclohexene monothiocarbonate)s (Scheme 11b) [162]. These copolymers contained at least 90% monothiocarbonate linkages. They had *T*_g_ = 112 °C, initial decomposition temperature of 214 °C, refractive index of 1.705, and might potentially be used as optical material. When using styrene oxide, copolymerization with COS proceeded with high regioselectivity via nucleophilic attack at methine carbon atom of oxirane with a formation of –(CHPhCH_2_SC(O)O)*_n_*– [163]. The results on catalytic copolymerization of oxiranes with COS were summarized and discussed in review of Luo, Zhang, and Darensbourg [164].

In contrast with Zn/Co DMCCs, the well-defined complex Co_3_[Co(CN)_6]2_ catalyzed copolymerization of PO with COS with a formation of poly(propylene monothiocarbonate)s (*M*_n_ = 66.4–139.4 kDa, *Ð*_M_ = 2.0–3.9) with suppressed oxygen–sulfur exchange reaction, the selectivity of the monothiocarbonate over carbonate linkages was >99%. The side product, cyclic thiocarbonate, was not formed [165].

#### 4.2.5. Copolymers of Two Different Oxiranes and CO_2_

Poly(cyclohexene carbonate) represents brittle plastic, which significantly limits its use. In recent study of Liu et al., transformation of this copolymer into elastomer was achieved via CO_2_/cyclohexene oxide/vinyl cyclohexene oxide terpolymerization followed by thiol–ene click reaction and Cu–S coordination post-functionalization. The resulting thermoplastic elastomers displayed good elastic recovery (remaining at 86% strength after five cyclic tests), a certain hardness (Shore A from 31 to 91), and adjustable mechanical properties [166].

#### 4.2.6. Functional Derivatives of PECs

**Scheme 12:**
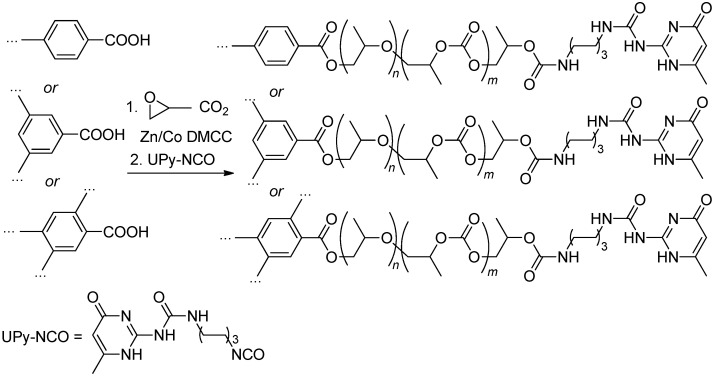
Synthesis and chain-end functionalization of PECs with different macromolecular architectures [152].

PECs, obtained by copolymerization of PO and CO_2_ with different molecular architectures, determined by the used initiators (benzenedi-, tri-, or tetracarboxylic acid), were functionalized by the product of reaction of 2-amino-4-hydroxy-6-methylpyrimidine with OCN(CH_2_)_6_NCO (Scheme 12); 0.1 mm thick films, prepared by compression molding, demonstrated different mechanical and adhesive properties, determined by copolymer architecture and microstructure [152]. The highest shear strengths *s* in lap joint tests was demonstrated by copolymer obtained using 1,3,5-benzenetricarboxylic acid as an initiator (*F*_CO2_ = 44%), the values of *s* were 7.5 MPa (stainless steel) and ~10 MPa (wood).

In light of future use of PEC polyols in polyurethane production, in 2024, Dong et al. proposed the use of primary aromatic amines as an initiators of PO/CO_2_ copolymerization, catalyzed by Zn/Co DMCC [167]. The ability of anilines to initiate copolymerization was determined by their *pK*_b_: 2-chloroaniline and 2-bromoaniline (*pK*_b_ = 11.35 and 11.47, respectively) were the most efficient initiators. Isomeric nitroanilines with close *pK*_b_ were inactive due to coordination of –NO_2_ group at catalytic centers. Low-MW amino PEC polyols, obtained using 4,4′-methylene-bis(2-chloroaniline) as an initiator, were suitable for the preparation of rigid polyurethane foams without any external catalyst.

**Scheme 13:**
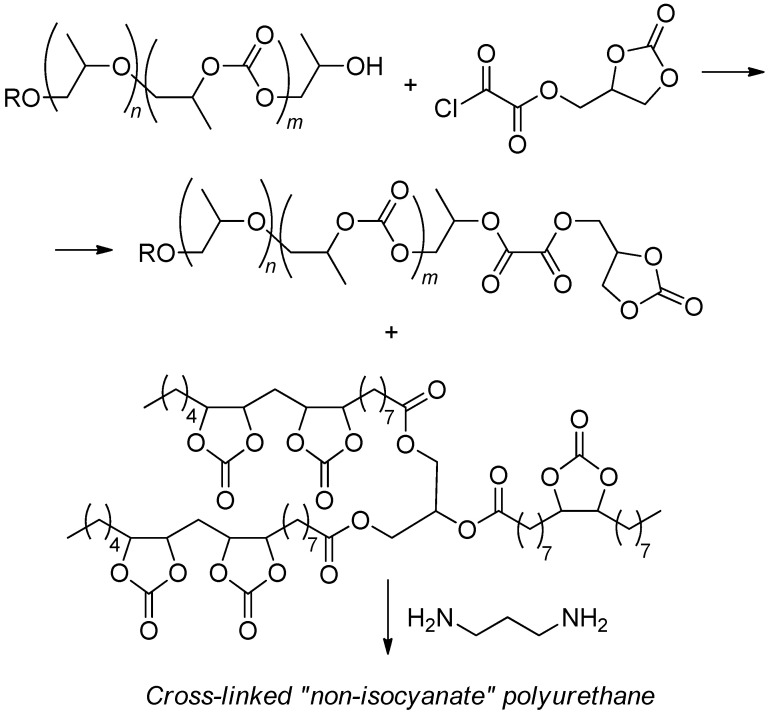
Synthesis of “non-isocyanate” polyurethane using functionalized PEC polyols [168].

An interesting and topical pathway of post-modification of PEC polyols, prepared using Zn/Co DMC catalysts, was the introducing of a cyclic carbonate end-group (Scheme 13). A copolymer modified in this manner was mixed with cyclic-carbonated soybean oil, and the mixture was then cross-linked by propane-1,3-diamine with a formation of “non-isocyanate” polyurethane [168].

### 4.3. Other Copolymers Based on Oxiranes

#### 4.3.1. Copolymerization of Oxiranes with Other Monomers

**Figure 17:**
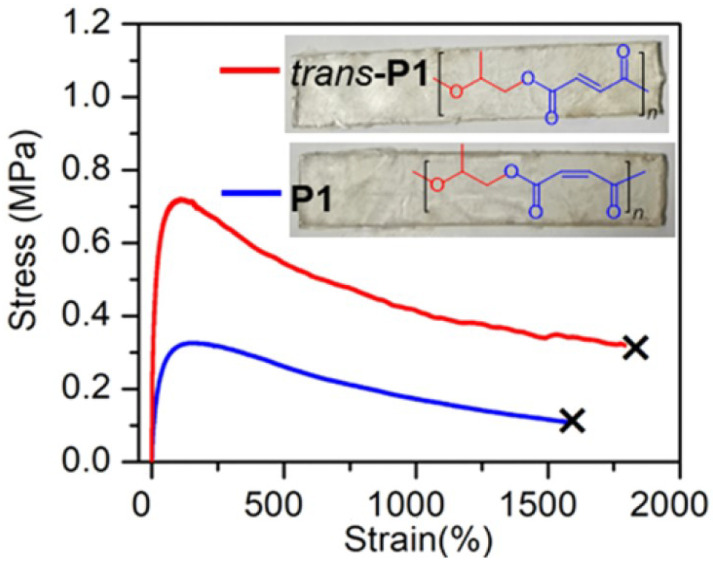
A marked difference in mechanical properties between *cis*-isomer of PO/MA copolymer obtained by alternating copolymerization in the presence of Zn/Co DMCC and its *trans*-isomer. Reprinted with permission from [170]. Copyright (2020) American Chemical Society, Washington, D.C., USA.

Introduction of reactive fragment into backbone of the macromolecule represent common and efficient approach to new materials; therefore, the combination of polyether fragments (contribution of oxirane) with unsaturated C=C bonds or functional groups from other comonomers appears to be promising, and it seems obvious to use DMC complexes as catalysts for the synthesis of similar copolymers. Back in 2004, PO was copolymerized with maleic anhydride in the presence of Zn/Co DMCC with a formation of alternating copolymer, productivity was up to 10 kg∙g_cat_^−1^, and remarkably, MA was not able to polymerize in the presence of this catalyst [169]. In 2020, similar copolymers attracted the attention of the Zhang’ group: they studied copolymerization of PO and other oxiranes (but-1-ene, hex-1-ene, and cyclohexene oxides, *tert*-butyl and phenyl glycidyl ethers) with MA, catalyzed by Zn/Co DMCCs [170]. In all cases, copolymers with <1 mol% of the ether content were obtained that had *M*_n_ = 47–152 kDa, high values of the temperature of decomposition *T*_d_ = 265.7–311.5 °C (5% weight loss), and high stress at break up to 25.3 MPa. Remarkable results were detected when studying copolymers of MA subjected to *cis*-/*trans*-isomerization using the previously published method [171]: mechanical characteristics (Figure 17) and biodegradability of the copolymer were significantly improved.

**Figure 18:**
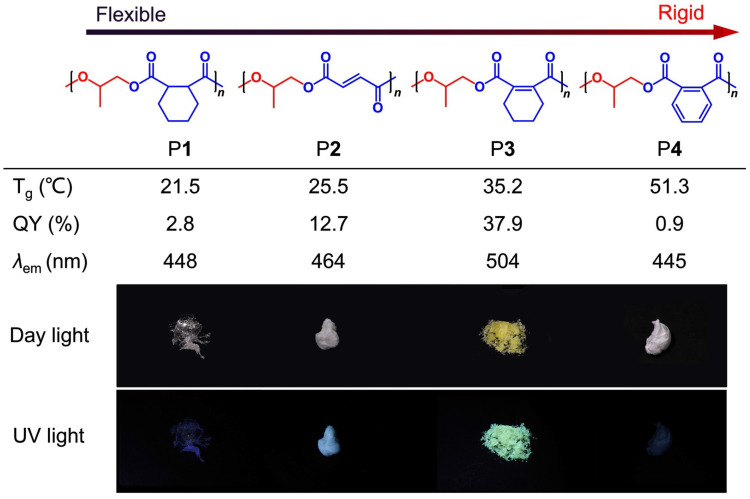
Chemical structures of P1, P2, P3, P4, listed as the increasing order of *T*_g_, indicative of the segmental mobility from flexibility to rigidity, summarized thermodynamic, and photophysical data and photographs of solid powders for four polyesters taken under daylight and UV light, respectively. *T*_g_: glass transition temperature; QY: quantum yield; λ_em_: photoluminescence emission maximum. Reprinted with permission from [172]. Copyright (2022) Wiley-VCH Verlag GmbH & Co., Weinheim, Germany.

Zn/Cr DMCC (~0.5 wt%) at 110 °C catalyzed copolymerization of PO with phthalic anhydride, and copolymer with *M*_n_ = 4.12 kDa and *Ð*_M_ = 1.29 was obtained [93], although this material was not studied in terms of mechanical and physical characteristics. But in 2022, Zhang et al. discovered the amazing property of similar linear non-conjugated polyesters: clusteroluminescence [172]. Four nearly alternating copolymers P1–P4 (Figure 18) were prepared using Zn/Co DMCC. The changes of *T*_g_ values indicated the decreasing segmental mobility from P1 to P4 at room temperature; copolymer P3 showed the maximum λ_em_ and the highest quantum yield (QY). The value of QY increased with an increase of the ester content in P3. The occurrence of intensive yellowish-green clusteroluminescence in P3 was explained under the assumption that forbidden n-π* transition of the carbonyl group is allowed in P3 and its analogs.

UV-vis spectral studies confirmed this assumption but revealed longer-wavelength emission peaks at 520–530 nm for P2 and P3, attributed to ester unit clusters, beyond the single C=O group level. From the point of view of application, clusteroluminescence quenching as a result of destruction of polyester clusters under the action of metal ions was illustrated by an example of Fe^3+^ ions: copolymer P3 has shown high selectivity to Fe^3+^, and therefore could be used as an efficient iron sensor.

**Figure 19:**
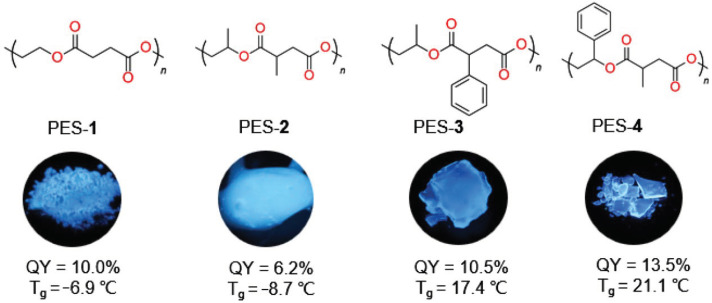
Chemical structures of PES-1 to PES-4, photographs of solid-state samples taken under 365 nm UV light, respectively, *T*_g_: glass transition temperature, QY: quantum yield. Reprinted with permission from [173]. Copyright (2022) Wiley-VCH Verlag GmbH & Co., Weinheim, Germany.

Further studies [173] of oxirane/anhydride-based polyesters, prepared using Zn/Co DMCCs, revealed clusteroluminescent materials with λ_em_ ~ 440 nm but with different emission efficiency (Figure 19).

**Scheme 14:**
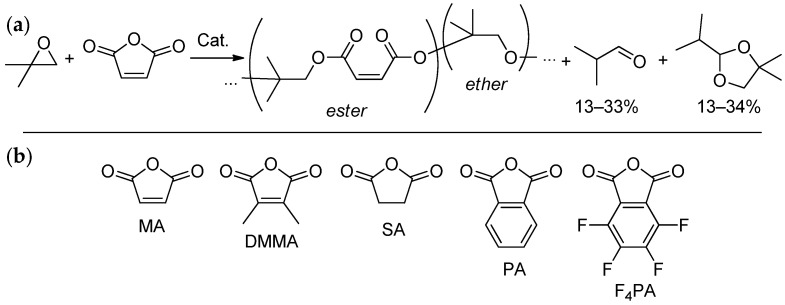
Copolymerization of isobutylene oxide with MA and formation of side products when using homogeneous catalysts (**a**); structures of anhydrides studied in copolymerization with isobutylene oxide in the presence of Zn/Co DMC complex (**b**) [174].

Copolymerization of isobutylene oxide and cyclic anhydrides was studied by Zhang et al. [174]. The screening of different catalysts in copolymerization with MA revealed significant flaws of different homogeneous catalysts manifesting in the formation of low-MW oligomers with high polyether content and side products, isobutyraldehyde and 2-isopropyl-4,4-dimethyl-1,3-dioxolane (Scheme 14a). When Zn/Co DMCC was used, high-MW polyesters with alternating microstructure (>99%) were obtained, and significant amounts of isobutyraldehyde were detected at 80–100 °C. When copolymerization reactions were conducted at 45–60 °C, 45–96% conversions were achieved after 6–9 h for MA, DMMA and PA (see Scheme 14b), copolymerization of SA and F_4_PA required 48 and 60 h to achieve 99% conversion. A 99% regioselectivity was observed for SA, PA and F_4_PA. Obtained copolymers represented semicrystalline polyesters (*T*_m_ = 67–141 °C) depending on the structures of anhydrides. The *cis*-/*trans*- isomerization of the C=C bond in the backbone of poly(IBO-*alt*-MA) was accompanied by a change of *T*_m_ from 72 to 153 °C.

The direct (i.e., statistical) copolymerization of cyclic siloxane monomers with oxiranes is impeded because of the insufficient nucleophilicity of the active alkoxide chain end of polyethers for the cleavage of a Si–O bond. Surprisingly, the Zn/Co DMC complex proved to be a capable catalyst for direct copolymerization of PO with (Me_2_SiO)_3_ at 120 °C [175]. During copolymerization, fast consumption of PO was observed during at the initial stage, whereas ROP of (Me_2_SiO)_3_ occurred when PO was consumed to nearly full conversion. As a result, gradient copolymers with *T*_g_ from −67 °C (3 mol% of Me_2_SiO) to −95 °C (46 mol% of Me_2_SiO) were formed.

#### 4.3.2. Copolymers of Oxiranes, CO_2_ and Anhydrides

The idea of the use of additional comonomers for improvement of mechanical characteristics of PCs and PECs or to create an opportunity for post-functionalization of copolymers has been investigated in numerous studies [6,176,177,178]. Preparation of cross-linkable copolymers via copolymerization of PO, CO_2_, maleic anhydride, and allyl glycidyl ether was conducted by Müller et al. in 2016, and allyl-substituted oxirane was introduced initially or at a late stage of copolymerization [179]. Subsequent UV curing with the use of (PhCO_2_)_2_ as an initiator resulted in transparent films of interest to coating applications.

**Scheme 15:**
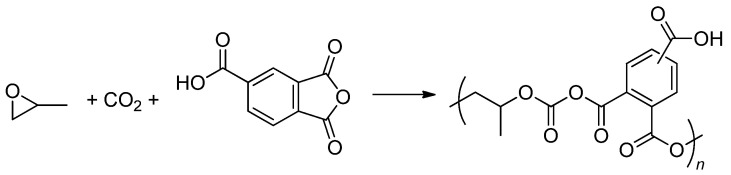
Copolymerization of propylene oxide, CO_2_ and trimellitic anhydride with a formation of alternating terpolymer [71].

A three-component catalyst comprising zinc glutarate, rare earth ternary complex, and Zn/Co DMC complex was proposed for the use in terpolymerization of PO, CO_2_, and trimellitic anhydride [71]. Under optimized conditions, the yield of copolymer was 66 g∙g_cat_^−1^. In large part, this copolymer had alternating microstructure (Scheme 15) and was characterized by *M*_w_ up to 83 kDa, unique thermal stability, and high decomposition temperature (10% loss of weight at 313 °C).

Terpolymerization of PO, CO_2_ and itaconic anhydride, catalyzed by Zn/Co DMCC (prepared using *^t^*BuOH as a CA), was complicated by isomerization of double bond in itaconic anhydride and cross-linking with a formation of gels [180]. The mechanism of cross-linking, presented in this work, seems not quite clear.

#### 4.3.3. Copolymers of Oxiranes, CO_2_ and Cyclic Esters

In 2015, the introduction of ε-caprolactone (ε-CL) units to a copolymer, containing ether and carbonate fragments, was proposed with the use of cross-chain exchange reaction (CCER) approach, a combination of two independent chain propagations generated by two different catalysts [181]; in this work, Zn/Co DMCC and tin(II) 2-ethylhexenoate (SnOct_2_) were applied for copolymerization of cyclohexene oxide, CO_2_, and ε-CL with a formation of materials that had wide spectrum of mechanical properties and optical characteristics.

Copolymers of PO, CO_2_, and *dl*-lactide (*dl*-LA), prepared in the presence of Zn/Fe DMCCs and synthesized by mechanochemical method in the presence of quaternary ammonium salts, had high *dl*-LA comonomer incorporation [83]. The further prospects of the use of similar catalysts seem doubtful due to very low catalytic activity (up to 5.65 g∙g_Zn_^−1^∙h^−1^).

Finally, note that the very idea of copolymerization of three types of comonomers with fundamentally different abilities to form metal–comonomer intermediates and mechanisms of ROP seems overly hopeful, bearing in mind diversity and sensitivity of the ROP coordination catalysts to the structure of the monomers [182].

### 4.4. Ring-Opening Polymerization of Cyclic Substrates Different from Oxiranes

Less strained (comparative to oxiranes) cycles including cyclic esters (lactones, lactides) were also studied in polymerization, catalyzed by DMCCs. In 2020, Liu et al. showed that Zn/Co nanolamellar DMCC (prepared using *^t^*BuOH as a CA [160]) in the presence of ~1 mol% of oxiranes initiates ROP of ε-CL in bulk at 120 °C with a formation of high-MW polymer (*M*_n_ up to 178 kDa) [94]. The results of this study are of interest considering that the ROP was efficiently initiated by cyclohexene oxide, whereas conventional ROP initiator BnOH was inactive. The following year [63], Kim et al. conducted a comparative study of ε-CL polymerization in the presence of Zn/Co DMCCs (0.33 mol% Zn), prepared with the use of ethyl acetoacetate, *^t^*BuOH, or Me_2_CHCN as CAs; the catalyst prepared with the use of Me_2_CHCN showed the highest productivity. Depending on the initiator used (ethylene glycol, propylene glycol, glycerol, or sorbitol), polymers with *M*_n_ = 0.75–4.0 kDa were obtained (SEC data), allowing them to be used in the preparation of poly(ester urethane) elastomers. Similar catalytic activities in polymerization of ε-CL and δ-valerolactone were demonstrated by Zn/Co DMCCs, prepared with the use of CH_3_NO_2_ or 1-methylpyrrolidin-2-one [65]. The effect of the CA on catalytic activity of Zn/Co DMCCs in ε-CL polymerization was also demonstrated in the comparative study of the complexes, prepared using different aliphatic nitriles that confirmed the highest efficiency of Me_2_CHCN [64].

Besides polyurethane production, low-MW ε-CL-based polyols (*M*_n_ = 1.0–4.2 kDa, *Ð*_M_ = 1.30–1.88), prepared with the use of Zn/Co DMCCs, were employed as soft segments to produce thermoplastic poly(ester–ester) elastomers containing poly(butylene terephthalate) as a hard segment [183].

### 4.5. Other Copolymers

**Scheme 16:**
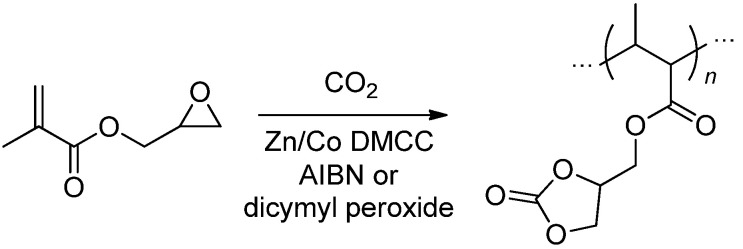
One-pot synthesis of poly(2-oxo-1,3-dioxolane-4-yl)methyl methacrylate [184].

The potential ability to fixation of CO_2_ by its interaction with oxiranes with a formation of cyclic carbonates (for more details see Section 4.6) allowed Liu et al. [184] to develop an original one-pot synthetic approach to poly (2-oxo-1,3-dioxolane-4-yl)methyl methacrylate, based on glycidyl methacrylate (Scheme 16). This approach was based on simultaneous use of a Zn/Co DMCC and [C_16_H_33_NMe_3_]Br catalytic system (0.3 mol% related to oxirane) and a free-radical initiator in DMF media at appropriate temperature. Up to 100 mol% of the carbonate content in polymers were achieved. UV-vis spectra of these copolymers showed high absorbance at 200–315 nm (UB-B and UV-C regions) and 93% transmittance (0.04 mm thick) for UV-A and visible regions, which makes these copolymers prospective UV-absorbing material for healthcare applications.

### 4.6. Synthesis and Transesterification of Cyclic Carbonates

#### 4.6.1. Synthesis of Cyclic Carbonates

Interaction of oxiranes with CO_2_ may lead to the formation of five-membered cyclic carbonates, this catalytic process attracts researchers’ attention as one of the possible ways of chemical fixation of CO_2_ with a formation of value-added products [185,186,187]. Zn/Co DMCCs, being efficient catalysts of copolymerization of oxiranes with CO_2_, can also be tuned to the formation of cyclic carbonates. In 2009, Park et al. reported the results of the study of addition of quaternary ammonium salt to Zn/Co DMCCs [188]. They showed that [R_4_N]X (R = *^n^*Pr, *^n^*Bu, *^n^*Hex, *^n^*Oct, *n*-C_12_H_25_; X = Cl or R = *^n^*Bu, X = Br) promote the formation of cyclic carbonates, 140 °C was the best reaction temperature condition at moderate pressure of CO_2_ (3.5 bar), and TON values up to 10^3^ and selectivity of 99% were achieved. In the presence of Zn/Co DMCC (3 wt%) and [*^n^*Bu_4_N]Br (1 wt%), 96% conversion of PO was achieved after 20 h at 60 °C and 20 bar of CO_2_ [189]. A continuation of Park’s group research [69] was an attempt to overcome the disadvantages of using two different catalysts (Zn/Co DMCCs and [R_4_N]X) by introducing of [C_16_H_33_NMe_3_]Br as a CA during the preparation of the catalyst. Under optimized conditions, a number of oxiranes were converted to corresponding cyclic carbonates (Table 4). The reaction mechanism, proposed in [69], does not hold water. The same catalytic system was successfully used for the synthesis of bis-carbonates from bis-epoxides containing two glycidyl fragments with different spacers [190].

When studying copolymerization of PO with CO_2_ using Zn/Co DMCCs, Zhang et al. observed that the trace amounts of water facilitate the formation of cyclic carbonates [191]. In their further study, they showed that binary catalytic systems, containing conventional Zn/Co DMCCs prepared using *^t^*BuOH and P123, in combination with quaternary ammonium salts, in the presence of water (260 ppm) provide formation of the corresponding cyclic carbonate with ~100% selectivity; the maximum TON was 6300 [51].

Zn/Fe DMCCs, prepared by ball milling at 140 °C and 30 bar of CO_2_ after 6 h of the reaction between PO and CO_2_, showed TON up to 2300, and the highest yield of propylene carbonate was 97.3% [80,81].

**Scheme 17:**
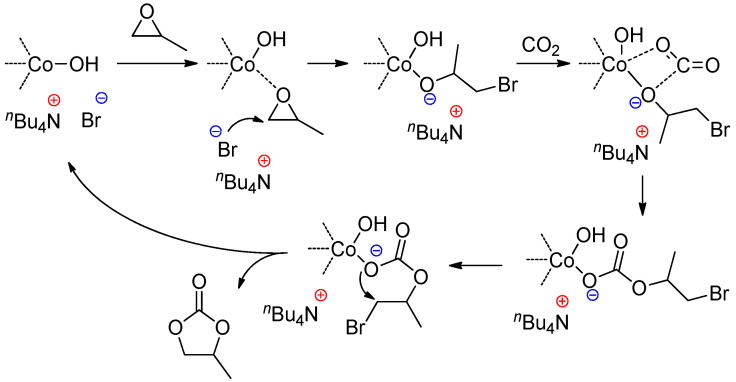
Proposed mechanism for CO_2_ and oxirane coupling catalyzed by the Co/Co DMCC—[*^n^*Bu_4_N]Br [192].

In 2022, the study of DMC M_3_[Co(CN)_6_]_2_ (M = Fe, Co) in combination with [*^n^*Bu_4_N]Br showed that Co/Co complex is a selective catalyst of the formation of cyclic carbonates [192]; the yields (1 mg of the catalyst, 25 mmol of oxirane, 65 °C, 10 bar, 7 h) exceeded 99% for PO, 1,2-butylene oxide and epichlorohydrin. The yields of carbonates significantly decreased with increasing of steric hindrance, as indicated by formation of 3-*tert*-butoxy-1,2-propylene carbonate, 1-phenyl-1,2-ethylene carbonate, and 3-phenoxy-1,2-propylene carbonate with yields of 93, 64, and 66%, respectively. The suggested mechanism of the reaction involves coordination of the oxirane oxygen atom at the Co Lewis acid site, nucleophilic attack of Br^−^ on the less sterically hindered C atom, coordination/insertion of CO_2_, and intramolecular cyclization (Scheme 17).

The same year, Znang et al. demonstrated that even Prussian blue Fe_4_[Fe(CN)_6_]_3_ mesoporous nanoparticles, surface-functionalized by –OH groups, provide quantitative yields of cyclic carbonates under mild reaction conditions (90 °C, 10 bar of CO_2_) [193].

Glycerol carbonate (4-hydroxymethyl-2-oxo-1,3-dioxolane) is one of the most valuable bifunctional building blocks in organic chemistry and polymer science [194,195]. Conversion of glycidol to glycerol carbonate is a difficult task, complicated by the formation of oligomeric and polymeric products. Comparative study of DMC complexes, prepared without the use of CAs, revealed the most active system, Zn_3_[Co(CN)_6_]_2_∙5H_2_O [66]. When using 0.02 mol% of the catalyst at 120 °C and 20 bar, 97% conversion of glycidol and 71% yield of glycerol carbonate were achieved.

#### 4.6.2. Transesterification of Cyclic Carbonates

**Scheme 18:**
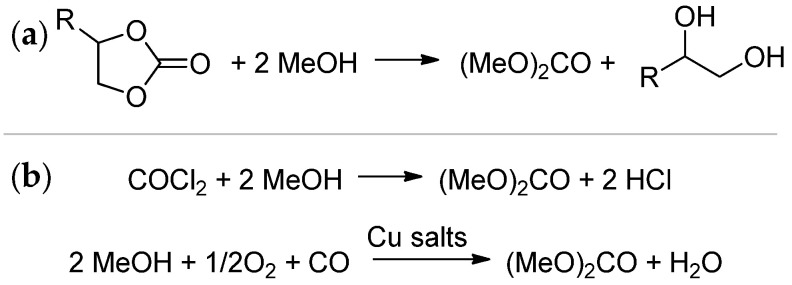
Methanolysis of cyclic carbonates (**a**) as an alternative to conventional methods of the synthesis of dimethyl carbonate (**b**).

Methanolysis of cyclic carbonates to produce dimethyl carbonate (Scheme 18a) is a green and atom-economical alternative to conventional method of the synthesis of (MeO)_2_C=O from phosgene; this approach can also compete with the Enichem catalytic process (Scheme 18b) [196,197].

In 2006, the use of Zn/Fe DMCC was proposed for methanolysis of propylene carbonate, isolated yield of (MeO)_2_C=O was 86% [198]. Further comparative study of different DMCCs (Zn, Mn, Ni/Fe and Zn, Mn, Ni/Co) [199] revealed the most efficient catalyst for this process, a Mn/Fe DMC complex, prepared by the reaction of K_4_Fe(CN)_6_ with MnCl_2_ at 1:8 molar ratio. At 140 °C and catalyst loading 5 g per mol of propylene carbonate, the TOF was 57.1 h^−1^ and selectivity of (MeO)_2_C=O formation was 96.1%. The loss of catalytic activity during the recycling process was 11.2% after four runs. The rate of ethylene carbonate conversion in the presence of Mn/Fe DMC was higher compared to propylene carbonate. For this catalyst and process, a simple two-step power low kinetic model was developed; good correlations with experimental data were observed [121].

### 4.7. The Use of DMCCs in Preparative Organic Chemistry

#### 4.7.1. Hydroamination

Hydroamination of terminal alkynes is of interest to organic chemistry, and DMCCs were studied in this reaction. For example, reaction of 4-isopropylaniline with PhC≡CH was catalyzed by Zn/Co DMCC prepared using poly(tetramethylene ether) glycol as a CA, and 99% yield of the hydroamination product was achieved after 24 h at 110 °C with 100 mg_cat_∙mmol^−1^ loading [41]. In a silica-supported Zn/Co DMC catalyzed reaction of 4-isopropylaniline with PhC≡CH in toluene at 110 °C, the TOF was only ~0.6 h^−1^ [73]. A well-defined DMC complex Zn_2_[Co(CN)_6_](OAc)∙4H_2_O showed higher catalytic activity in hydroamination of PhC≡CH by 2,6-diisopropylaniline (TOF = 0.87 h^−1^) in comparison with conventional Zn/Co DMCCs [60]. A series of Zn/Co DMCCs, prepared with the use of different CAs and co-CAs, also showed moderate activities; aliphatic amines have proven to be less reactive [40].

Recently performed experiments on Zn/Co DMC-catalyzed hydroamination of substituted phenylacetylenes by substituted anilines confirmed low productivity of these catalysts; at 5 wt% loadings, the yields of the corresponding imines were up to 94% after 12 h at 110 °C [200]. The same yields were achieved in the reaction of PhNHNH_2_ with PhC≡CH.

#### 4.7.2. Reductive Amination

Zn/Co DMCC, prepared using *^t^*BuOH and PEG-10000 and pre-activated at 180 °C, demonstrated moderate activity in reductive amination of ketones and aldehydes by substituted anilines; BnNH_2_ (10 wt% of the catalyst, 60 °C, 5 h, MeOH as a solvent), poly(methylhydrosiloxane) was used as a reducing agent, and the yields of amines amounted to 52–98% [201]

#### 4.7.3. Transesterification

Fatty acid methyl esters (FAMEs) are intermediates for the production of fine oleochemicals; mostly FAMEs are considered as a fuel (biodiesel) [202]. Industrial production of FAMEs via transesterification of natural oils and fats is based on the use of homogeneous catalysts; however, heterogeneous systems keep attracting the attention of researchers due to the ease of separation and recyclability [203,204,205,206]. In 2020, Zn/Fe DMCC was studied in methanolysis of jatropha oil; 95% conversion was achieved at [MeOH]/[Oil] = 10:1, 160 °C and 5 wt% catalyst loading [207].

#### 4.7.4. Acid-Nitrile Exchange

**Scheme 19:**
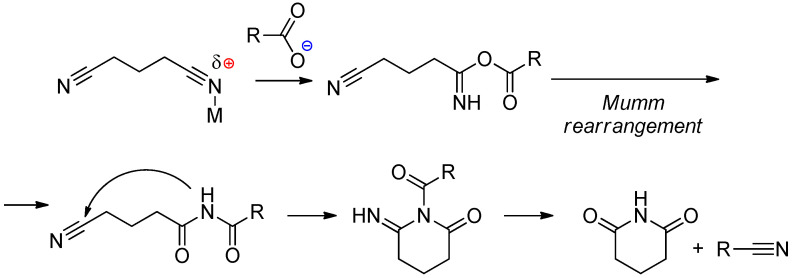
DMC-catalyzed acid-nitrile exchange reaction [208].

The synthesis of nitriles from carboxylic acids can be performed by a thermodynamically-driven reaction between acid and glutaronitrile (Scheme 19). This thermally induced process proceeds without any additional catalyst with ~50% yields (2-phenylbutyric acid as a substrate, 215 °C, 16 h), but some inorganic materials including DMC complexes can accelerate the reaction. The best results among other DMCCs was demonstrated by Co(II)–Co(III) system (86%), and homogeneous catalyst AlCl_3_ turned out to be equally active (90% yield); the catalyst loadings were 10 mol% [208].

#### 4.7.5. Synthesis of Phosphoramidates

**Scheme 20:**
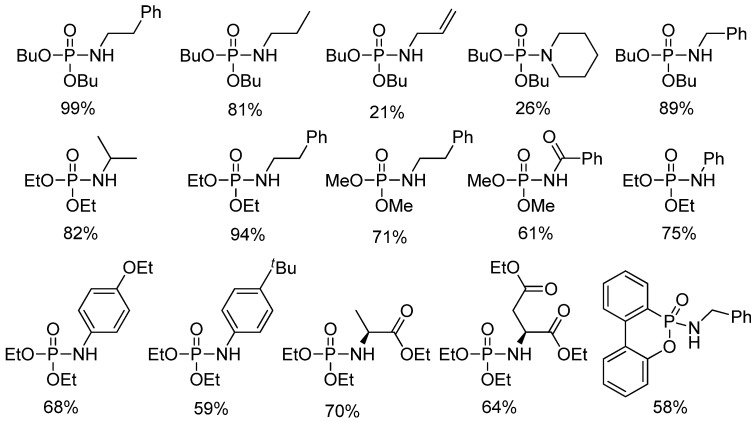
Molecular structures of phosphoramidates prepared using Cu_3_[Co(CN)_6_]_2_-based DMCC; the yields of the products based on dialkyl phosphites are given [209].

Cu_3_[Co(CN)_6_]_2_-based DMCC in the presence of I_2_ as an additive proved capable of catalyzing oxidative transformation of phosphites to phosphoramidates [209]. After optimization of the reaction conditions for (*^n^*BuO)_2_P(O)H/PhCH_2_CH_2_NH_2_ substrates (3 wt% of the catalyst, 30 min, 20 °C, 1:2:0.15 phosphite/amine/I_2_ molar ratio in CH_2_Cl_2_ under O_2_ atmosphere), the method was applied to different phosphites and amines (Scheme 20). Catalytic amounts of I_2_ provided in situ formation of iodophosphate intermediate, followed by the formation of the P-N bond; the catalytic cycle was closed by aerobic oxidation involving the active center of DMC complex.

#### 4.7.6. Epoxidation

The complex Cu_3_[Co(CN)_6_]_2_ (0.6 mg∙mmol^−1^) was studied as a catalyst of epoxidation of styrene, cyclohexene, and norbornene using *^t^*BuOOH as an oxidant; high selectivity of the formation of oxirane was achieved only for norbornene. In the case of styrene up to 64% of PhCHO were formed, depending on the reaction solvent [88].

Similar results in epoxidation of styrene by *^t^*BuOOH were obtained for wide spectrum of DMC complexes (Co(II)–Fe(III), Co(II)–Fe(II), Co(II)–Co(III), Cu(II)–Fe(III), Ni(II)–Fe(III), Fe(II)–Co(III), Fe(II)–Fe(III), and Fe(II)–Fe(II)), with and without modification by ionic liquids [210]. The complex Co_3_[Fe(CN)_6_]_2_ (6 mg∙mmol^−1^), modified with 1-butyl-3-methylimidazolium bis(trifluoromethylsulfonyl)imide showed the highest catalytic efficiency (90% conversion after 6 h at 72 °C), but the reaction products contained 35 mol% of PhCHO.

Modification of the structure of Fe_3_[Co(CN)_6_]_2_ by introducing of ROH (R = C1–C6 alkyl) failed to reach any breakthrough in epoxydation of styrene; a slight increase of activity was accompanied by decrease of selectivity of the formation of oxirane [211].

#### 4.7.7. Catalytic Rearrangements of Biomass-Derived Furans

**Scheme 21:**
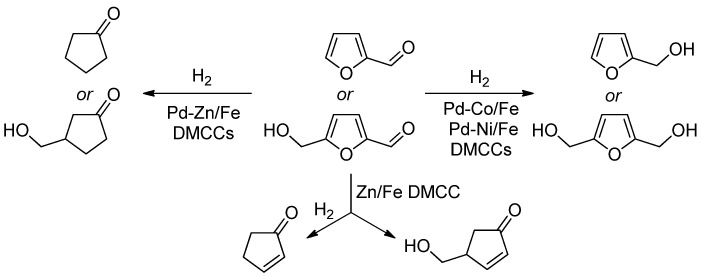
Synthesis of value-added chemicals from furfural and hydroxymethylfurfural [78,212].

Hydrogenative ring-rearrangement of biomass-derived furfural and 5-hydroxymethyl furfural to other value-added products was the subject of the study of Deng et al. [78]. They showed that Pd-Zn/Fe DMCCs demonstrate high efficiency for the synthesis of cyclopentanone or 3-hydroxymethyl cyclopentanone with yields up to 96.6 and 87.5%, respectively, whereas the Pd-Ni/Fe and Pd-Co/Fe DMCCs with a weak Lewis acidity provide ~90% yields of furfuryl alcohol or 2,5-bis(hydroxymethyl)furan, respectively. The catalyst loadings were ~10 mg∙mmol^−1^ (150 °C, 40 bar of H_2_, aqueous media), Pd-Zn/Fe DMCCs demonstrated excellent reusability after simple washing by water. In the absence of H_2_, Zn/Fe DMC complex efficiently catalyzed transformation of 5-hydroxymethyl furfural to corresponding cyclopentenone (Scheme 21); it is noteworthy that in the presence of H_2_, furfural transformed to cyclopent-2-enone [212]. The authors proposed that Zn atoms represent acid and hydrogenation active sites, i.e., Zn/Fe DMCC is a rare example of Zn-based hydrogenation catalyst.

Conversion of furane derivatives to cyclopentanones and cyclopentenones represents a promising “green” alternative to conventional decarboxylative ketonization of adipic acid. Comparison of different catalytic systems, studied in conversion of furfural and 5-hydroxymethyl furfural to cyclopentanones [213], revealed the undoubted advantage of Pd-Zn/Fe DMCCs, which were inferior in selectivity only to Au/TiO_2_ catalyst. In this way, DMCCs can be used to produce highly valuable furan-based chemicals from biomass, and this direction is promising and worthy of further development.

## 5. Conclusions and Prospects

**Scheme 22:**
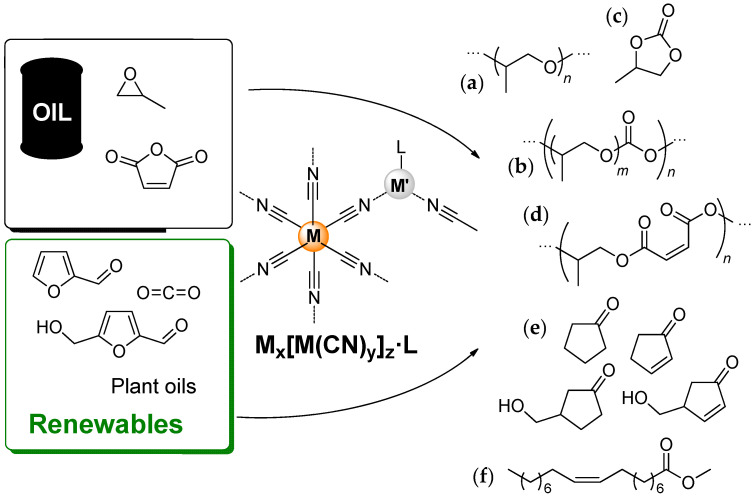
The current use and prospective applications of DMCCs: synthesis of polyethers (**a**); poly(ethers-*co*-carbonate)s (**b**); cyclic carbonates (**c**); copolymers of oxiranes with anhydrides (**d**); cyclopentanones and cyclopentenones from biobased furans (**e**) and methyl oleate and related esters from plant oils (**f**).

The current use and prospective applications of DMCCs are presented in generalizing Scheme 22, which also reflects the type of the raw material, fossil or renewable. Among other DMCCs, Zn/Co DMC complexes remain the most active and widely used catalysts for the production of relatively low-MW polyols, products of ROP of propylene oxide (Scheme 22a), and other oxiranes to a lesser extent. Chemical fixation of CO_2_ in polyether backbone meet the requirements of contemporary green agenda and allows obtaining poly(ethers-*co*-carbonate)s (Scheme 22b), polyols no less in demand. Both copolymers are widely used in polyurethane industry. A deeper understanding of the mechanism of the action of DMCCs allows developing active catalysts with new formulations, morphologies, and properties; however, other DMC complexes distinct from Zn/Co DMCCs are not yet ready to compete with the latter in these industrially implemented processes. The mechanism of the catalytic action of DMC complexes in the processes, presented in Scheme 22a,b, is still not clear. Theoretical and fundamental studies are based on idealized models, and both mononuclear and binuclear concepts are still being discussed. Hopefully, the current progress in quantum chemical modeling and physico-chemical monitoring of the catalytic processes will clarify the mechanisms of the action of DMC complexes in the near future.

As stated above, an alternative method of chemical fixation of CO_2_ is a transformation of oxiranes to cyclic carbonates (PCs), highly demanded solvents and reagents (Scheme 22c). In this reaction, DMC complexes of other metals, distinct from conventional Zn/Co DMCCs, can be used. At the same time, development of new types of Zn/Co DMCCs with new complexing agents or via deeper design of the catalyst’s morphology (layered materials) also open up new growth avenues.

Besides preparation of polyols, PECs and PCs, DMCCs are increasingly used in the synthesis of new types of polymeric materials (Scheme 22d) promising for their applications as adhesives, elastomers, sensors, and UV-absorbing materials. Currently, there is a growing interest in the use of DMCCs in fine organic chemistry, and their applications for (hydro)amination, transesterification, and catalytic processing of renewable raw materials such as biobased furan derivatives (Scheme 22e) and triglycerides (Scheme 22f). As can be seen from data presented in Section 4.7, the catalytic potential of DMCCs in synthetic organic chemistry and biomass valorization is still not fully disclosed. These green, sustainable, and efficient processes need be further explored in the future.

## Figures and Tables

**Table 1:** PO polymerization results of the prepared DMC catalysts [62] (for catalyst legends, see Scheme 3) ^1^.

Entry	Catalyst	Init. Time, min	Cat. Longevity, min	Polymer Yield, g	TOF, ([PO]∙ [Zn]^−1^∙h^−1^)	Unsatur. Level ^2^	*M*_n_, kDa (*Ð*_M_)
1	Benchmark DMC	1	29	27.5	132,000	0.004	5.69 (4.88)
2	[DMC-Cl][MeOCMe_2_OH]	heating	30	27.9	120,000	0.024	6.05 (1.18)
3	[DMC-Cl][tBuOH]	4	29	28.4	126,000	0.022	6.17 (1.16)
4	DMC-OCMe_2_OMe	3	25	23.0	119,000	0.004	2.62 (3.70)
5	DMC-(OCMe_2_OMe/Cl)(0.25/0.75)	2	32	31.5	127,000	0.018	5.33 (1.40)
6	DMC-(OCMe_2_OMe/Cl)(0.5/0.5)	3	30	29.9	129,000	0.014	6.11 (3.57)
7	DMC-(OCMe_2_OMe/Cl)(0.75/0.25)	2	27	26.2	125,000	0.006	5.44 (2.00)
8	DMC-OtBu	heating	27	26.6	127,000	0.004	6.07 (7.17)
9	DMC-(OtBu/Cl)(0.5/0.5)	7	31	28.7	120,000	0.005	6.40 (6.44)
10	DMC-OAc (60 °C, evacuation)	2	29	30.0	134,000	0.016	6.91 (2.53)
11	Zn_2_[Co(CN)_6_](OAc)·4H_2_O	1	28	27.3	126,000	0.014	6.22 (11)
12	DMC-(OAc/OtBu)(0.95/0.08)	0	30	31.9	137,000	0.013	7.15 (1.75)
13	DMC-(OAc/OtBu)(0.68/0.31)	0	29	29.6	132,000	0.010	7.06 (4.02)
14	DMC-(OAc/OtBu)(0.42/0.57)	4	28	28.2	130,000	0.007	6.48 (10.7)
15	DMC-(OAc/OtBu)(0.31/0.71)	heating	24	24.9	134,000	0.005	6.10 (9.12)
16	DMC-(OAc/OtBu/Cl)(0.68/0.38/0.06)	0	29	29.9	133,000	0.010	6.94 (4.17)
17	DMC-(OAc/OtBu/Cl)(0.74/0.24/0.10)	1	29	30.1	134,000	0.008	6.30 (3.08)
18	DMC-(OAc/OtBu/Cl)(0.59/0.38/0.15)	0	29	30.0	134,000	0.007	6.56 (3.32)
19	DMC-(OAc/OtBu/Cl)(0.41/0.43/0.28)	1	28	29.2	134,000	0.005	6.28 (3.73)
20	DMC-(OAc/OtBu/Cl)(0.34/0.39/0.39)	2	31	31.0	129,000	0.006	6.58 (3.42)

^1^ Polymerization conditions: DMC (4.0 μmol Co, 1.6−1.9 mg), PPG starter (*M*_n_ = 425 Da, 4.25 g), heating to 125 °C, feeding 5.0 mL PO for 4 min for initiation, waiting until almost full consumption of PO (initiation time), continuous feed of PO (1.25 mL∙min^−1^) at 130−145 °C with spontaneous temperature control. ^2^ Calculated based on ^1^H NMR spectra.

**Table 2:** PO/CO_2_ copolymerization results of the prepared DMC catalysts [62] (for catalyst legends, see Scheme 3) ^1^.

Entry	Catalyst	Init. Time, min	Polymer Yield, g	TOF, [PO]∙ [Zn]^−1^∙h^−1^; [CO_2_]∙ [Zn]^−1^∙h^−1^	Cyclic Carbonate, wt% ^2^	*F*_CO2_, mol% ^3^	*M*_n_, kDa (*Ð*_M_)
1	Benchmark DMC	36	25.4	19,900; 2980	2.5	15	5760 (3.34)
2	[DMC-Cl][MeOCMe_2_OH]	54	5.6	4070; 1060	25	26	1420 (3.04)
3	[DMC-Cl][tBuOH]	25	3.1	2280; 547	16.2	24	1120 (1.78)
4	DMC-OCMe_2_OMe	191	3.4	2640; 422	9.2	16	1270 (1.55)
5	DMC-OtBu	111	25.9	20,200; 3030	1.3	15	6250 (4.04)
6	DMC-(OCMe_2_OMe/Cl)(0.5/0.5)	42	6.7	4930; 1180	16	24	1630 (2.07)
7	DMC-(OtBu/Cl)(0.5/0.5)	37	21.7	16,500; 3130	2.4	19	5450 (8.81)
8	DMC-OAc (60 °C, evacuation)	39	18.7	14,800; 1920	4.9	13	3960 (1.76)
9	Zn_2_[Co(CN)_6_](OAc)·4H_2_O	45	6.8	4850; 1410	7.4	29	1960 (1.73)
10	DMC-(OAc/OtBu)(0.95/0.08)	13	26.4	18,500; 5910	3.5	32	5420 (1.43)
11	DMC-(OAc/OtBu)(0.68/0.31)	15	28.4	20,800; 5190	4.0	25	3380 (5.39)
12	DMC-(OAc/OtBu)(0.42/0.57)	31	19.7	14,400; 3600	3.4	25	5970 (4.20)
13	DMC-(OAc/OtBu)(0.31/0.71)	49	19.3	11,500; 3040	2.4	21	4810 (9.32)
14	DMC-(OAc/OtBu/Cl)(0.68/0.38/0.06)	21	24.0	17,800; 4090	2.7	23	5510 (5.41)
15	DMC-(OAc/OtBu/Cl)(0.74/0.24/0.10)	17	25.8	19,000; 4560	1.1	24	5790 (3.33)
16	DMC-(OAc/OtBu/Cl)(0.59/0.38/0.15)	15	26.3	19,500; 4480	1.8	23	5830 (2.63)
17	DMC-(OAc/OtBu/Cl)(0.41/0.43/0.28)	22	25.6	19,100; 4200	2.8	22	6070 (2.71)
18	DMC-(OAc/OtBu/Cl)(0.34/0.39/0.39)	14	23.0	17,500; 3320	3.2	19	5510 (3.28)
19 ^4^	DMC-(OAc/OtBu)(0.95/0.08)	20	18.2	12,000; 5040	3.7	42	4290 (1.43)
20 ^5^	DMC-(OAc/OtBu)(0.95/0.08)	7	22.2	15,400; 5100	3.5	33	4960 (1.86)
21 ^6^	DMC-(OAc/OtBu)(0.95/0.08)	13	25.3	34,100; 10,900	3.4	32	5420 (1.43)
22 ^7^	DMC-(OAc/OtBu)(0.95/0.08)	6	5.4	3480; 1600	3.2	46	35.50 (7.17)

^1^ Polymerization conditions: DMC (11 μmol-Co, 4.5–5.2 mg), PPG starter (*M*_n_ = 425 Da, 4.25 g), heating to 110 °C, charging CO_2_ to 30 bar, feeding 5.0 mL PO for 4 min for initiation, waiting until pressure drops from 32 to 30 bar (initiation time), continuous feed of PO at 0.50 mL∙min^−1^ for 54 min with temperature control using external heating, charging CO_2_ to 30 bar whenever pressure drops to 25 bar. ^2^ (Propylene carbonate mass)/(polymer mass + propylene carbonate mass) determined on ^1^H NMR spectra. ^3^ (Carbonate linkage)/(ether linkage + carbonate linkage) in polymers excluding cyclic carbonates determined on ^1^H NMR spectra ^4^ Polymerization temperature of 100 °C. ^5^ Pressure of 40–35 bar. ^6^ Two times higher PO feeding rate (1.0 mL/min) and half-times shortened reaction time (28 min). ^7^ Copolymerization was performed by replacing the starter with THF (4.25 g) at 100 °C with the PO feed rate of 1.0 mL/min for 28 min.

**Table 3:** Comparison of the performance of the synthesized transition metal hexacyanoferrate(II) complexes with the performance of other hexacyanometallate catalysts [87] ^1^.

Catalyst	TON ^2^	TOF ^3^	F_CU_ ^4^, mol%	W_PC_ ^5^, wt%	S_CO2_ ^6^, %	S_PO_ ^6^, %	R_PEC_ ^7^, %	*M*_w_, kDa	*Ð*_M_
Ni_2_[Fe(CN)_6_]	93	4	14.9	1.4	94.2	99.1	92.4	3.6	5.4
Co_2_[Fe(CN)_6_]	223	9	29.3	5.4	88.2	96.2	67.0	20.2	4.0
KFe[Fe(CN)_6_]	170	7	13.5	19.8	46.6	86.6	85.3	14.0	5.1
Zn_2_[Fe(CN)_6_]	178	7	19.8	7.5	76.7	95.0	83.8	3.4	4.4
Ni_3_[Co(CN)_6_]_2_	86	4	22.3	0.4	98.9	99.7	79.5	11.8	10.5
Co_3_[Co(CN)_6_]_2_	544	23	20.0	4.2	87.4	97.2	71.9	68.6	4.1
Fe_3_[Co(CN)_6_]_2_	428	18	16.3	8.4	73.6	94.5	75.1	85.4	6.3
Ni_3_[Fe(CN)_6_]_2_	84	4	24.0	0.6	98.4	99.6	73.9	11.7	15.8
Co_3_[Fe(CN)_6_]_2_	296	12	33.5	13.3	75.4	90.2	66.0	50.0	5.9
Fe_4_[Fe(CN)_6_]_3_	162	7	17.3	43.1	26.2	67.2	83.3	6.0	8.4
Zn_3_[Co(CN)_6_]_2_	1279	53	9.7	8.9	61.9	94.4	86.6	8.3	2.5

^1^ Polymerization conditions: 24 h at 90 °C and 20 bar of CO_2_. Catalysts concentration = 2500 ppm, PO volume = 50 mL. ^2^ In mol_PO_∙mol_M(II)_^−1^. ^3^ In mol_PO_∙mol_M(II)_^−1^∙h^−1^. ^4^ Carbonate unit content, molar fraction of CO_2_ in the polymer. ^5^ Weight fraction of propylene carbonate. ^6^ Selectivities of CO_2_ and PO, calculated based on ^1^H NMR spectral data [86]. ^7^ The poly(ether-carbonate)-to-polycarbonate linkage ratio, calculated based on ^1^H NMR spectral data [157].

**Table 4:** Cycloaddition of CO_2_ and various oxiranes, catalyzed by Zn/Co DMC complex, prepared using [C_16_H_33_NMe_3_]Br as a complexing agent [69] ^1^.

Entry	Oxirane	Cyclic Carbonate	Time, h	Conv., %	Yield, %	TON ^2^
1	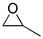	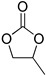	6	82	82	3075
2	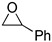	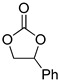	6	93	92	3486
3	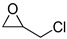	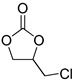	6	95	91	3563
4	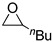	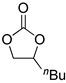	6	86	83	3225
5	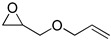	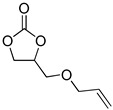	4	92	91	3450
6	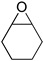	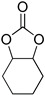	12	47	46	1763

^1^ Reaction conditions: oxirane = 18.6 mmol, DMCC amount = 20 mg, *p*(CO_2_) = 12 bar. ^2^ Turnover number (TON): mol of oxirane converted per mol of Zn.
